# Engineering Gene Networks to Emulate *Drosophila* Embryonic Pattern Formation

**DOI:** 10.1371/journal.pbio.0030064

**Published:** 2005-02-22

**Authors:** Mark Isalan, Caroline Lemerle, Luis Serrano

**Affiliations:** **1**European Molecular Biology Laboratory, Structures and BiocomputingHeidelbergGermany; Cambridge UniversityUnited Kingdom

## Abstract

Pattern formation is essential in the development of higher eukaryotes. For example, in the *Drosophila* embryo, maternal morphogen gradients establish gap gene expression domain patterning along the anterior-posterior axis, through linkage with an elaborate gene network. To understand the evolution and behaviour of such systems better, it is important to establish the minimal determinants required for patterning. We have therefore engineered artificial transcription-translation networks that generate simple patterns, crudely analogous to the *Drosophila* gap gene system. The *Drosophila* syncytium was modelled using DNA-coated paramagnetic beads fixed by magnets in an artificial chamber, forming a gene expression network. Transient expression domain patterns were generated using various levels of network connectivity. Generally, adding more transcription repression interactions increased the “sharpness” of the pattern while reducing overall expression levels. An accompanying computer model for our system allowed us to search for parameter sets compatible with patterning. While it is clear that the *Drosophila* embryo is far more complex than our simplified model, several features of interest emerge. For example, the model suggests that simple diffusion may be too rapid for *Drosophila*-scale patterning, implying that sublocalisation, or “trapping,” is required. Second, we find that for pattern formation to occur under the conditions of our in vitro reaction-diffusion system, the activator molecules must propagate faster than the inhibitors. Third, adding controlled protease degradation to the system stabilizes pattern formation over time. We have reconstituted transcriptional pattern formation from purified substances, including phage RNA polymerases, ribonucleotides, and an eukaryotic translation extract. We anticipate that the system described here will be generally applicable to the study of any biological network with a spatial component.

## Abstract 2

To understand how patterns are established during early development, these authors have created an artificial system to mimic aspects of the early *Drosophila* embryo

## Introduction

Engineering a system to emulate a particular behaviour can be an extremely informative approach to systems biology [1,2,3,4,5]. Even if the natural biochemical interactions are well characterized, it remains a considerable challenge to reconstruct a physical system with the appropriate behaviour. Step-by-step reconstruction allows theoretical assumptions and models to be refined. Not only is complexity reduced by removing the context of the whole organism, but by reconstitution of pattern formation from purified substances (in this case, RNA polymerases, ribonucleotides, and a translation extract) the sufficiency of a proposed mechanism of pattern formation can be demonstrated.

In this work, our primary aim was the development of an in vitro system that allows the careful buildup of complex networks under controlled conditions. To demonstrate the usefulness of such a system, we decided to reconstruct a developmental pattern-formation program based on the formation of a gradient of a transcription activator—a “morphogen”—and to link it to a network of transcription repressors. In a sense, we set out to design a patterning system similar to chemical reaction-diffusion systems (see below). However, the use of components such as transcription activators and repressors in an in vitro transcription-translation system made the system significantly closer to a biological system, albeit highly simplified when compared to the complexity of eukaryotic transcription [6].

Patterning systems can be thought of as belonging to one of two principal types: First, there are systems with homogeneous initial conditions that self-organise after early random symmetry-breaking events ([7,8]; for a biological example, *Fucus,* see [9]). Second, there are systems with initial localisation of the components, which can form concentration gradients of activities from their respective sources [10,11].

The first class of patterning system, involving reaction-diffusion from initially homogeneous conditions, was first proposed by Turing in 1952 [7] and was further developed by Meinhardt and Gierer in the 1970s, in their model of patterning with short-range autoactivator and long-range lateral inhibitor [8]. Although there are many likely biological candidates for such activator-inhibitor systems (reviewed in [12]), none has been reengineered from first principles. In contrast, significant progress has been made in reconstituting purely chemical reactions that self-organise, such as the Belousov-Zhabotinski reactions [13,14,15] and Turing-type, Meinhardt-Gierer (M-G), and oscillatory reactions [16,17,18,19,20]. In a more biological context, similar spatiotemporal patterns, consisting of propagating concentration waves, have been modelled for a glycolytic enzyme oscillator in yeast [21].

In the second class of patterning system, where the initial conditions are nonhomogeneous, patterning begins with an asymmetry: a localised source of morphogen (shape-defining molecules that form a concentration gradient and function in a concentration-dependent manner so as to determine positional information in a patterning field [22]; reviewed in [23,24,25]).

The question of how morphogen gradients are formed and maintained is still a matter of keen debate and study [26,27,28], with many proposed mechanisms (reviewed in [29,30]). In the simplest case, a stable gradient could be formed by passive diffusion [31] and uniform degradation, although it has been suggested that enhanced morphogen degradation near the source leads to increased robustness against morphogen fluctuations during patterning [32]. Also, “ligand trapping” by the receptor for a morphogen can have significant effects on the shape of a morphogen gradient [33], as in the case of Torso diffusing in the extracellular space surrounding the *Drosophila* oocyte [34].

In most cases in metazoa, morphogens define patterns over fields of many cells (reviewed in [30,35]), but there is one special case in embryonic development that has been particularly well studied, in which a morphogen gradient operates in a single-celled, multinuclear syncytium: the early *Drosophila* embryo [10,11]. In this system, early patterning is mediated by maternal morphogen factors, which are thought to diffuse and form gradients to guide patterning within a large multinuclear cell (reviewed in [36,37,38]). After egg deposition, an embryo forms a segmentation pattern within 3 h, under the influence of a hierarchical sequence of gene expression interactions involving gap genes, pair-rule genes, and segment polarity genes [39]. Maternal elements—in particular, the morphogen Bicoid—guide this process, setting distinct initial conditions.

**Figure 1:**
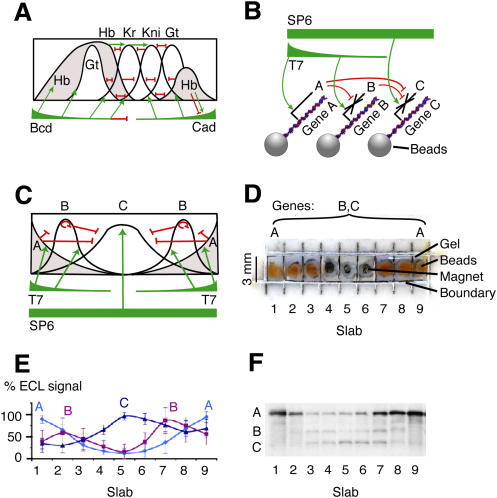
Gene Circuits and Chambers (A) Principal interactions in the *Drosophila* gap gene network, modelled after [37]. Relative levels and distributions of Hunchback (Hb), Giant (Gt), Krüppel (Kr), Knirps (Kni), Bicoid (Bcd), and Caudal (Cad) shown from anterior (left) to posterior (right). Green arrows indicate activation, red T-bars repression. (B) Artificial gene network design, with transcription activators T7 and SP6 polymerases, and zinc finger repressors A, B, and C. Genes are immobilised on paramagnetic beads, and T7 forms a directional concentration gradient. (C) Principal interactions in a simple designed network. (D) Transcription-translation chamber. Genes for repressor A are localised at the “poles,” whereas B and C are ubiquitous. Gel slabs 4–6 have been excised, exposing the magnets below, illustrating gel dissection for Western blot analysis. (E) Normalised Western data for four replicate chambers, showing mean levels of A, B, and C after 20 min (± One standard deviation). (F) Sample Western blot from the four-replicate experiment.

Work by Driever and Nüsslein-Volhard [10,11] demonstrated that Bicoid protein possessed three characteristics of a classic morphogen: (i) a localised source of cytoplasmic activity (through *bicoid* RNA transport to the anterior pole of the egg, involving microtubules and maternal genes [40,41,42,43,44]); (ii) formation of a concentration gradient from the source; and (iii) concentration-dependent activity that determines positional information within the gradient (reviewed in [38]). Bicoid has at least two functions that contribute to its function as a morphogen: transcription activation and translation inhibition. Acting as a transcription factor, Bicoid can activate a number of downstream gap genes, including *hunchback, knirps, giant,* and *Krüppel,* whose products cross-react in a complex and mainly repressive interaction network to modulate each other's expression (reviewed in [37]; modelled in [45,46]). However, Bicoid does not simply function independently as a morphogen at the top of the gap gene hierarchy. Although Bicoid is responsible for anterior expression of zygotic Hunchback [47], it actually requires maternally expressed Hunchback as a cofactor to function anteriorly [48]. Meanwhile, in the posterior, the maternal *hunchback* mRNA is initially translationally repressed by the posterior determinant Nanos [49]. Moreover, the terminal gap genes *tailless* and *huckebein* are activated independently but serve to repress zygotic gap gene expression at the poles of the embryo, thus influencing patterning [50,51,52]. In its other role, as a translation inhibitor of *caudal* mRNA, Bicoid initially inhibits the uniformly expressed mRNA to form a concentration gradient of the protein Caudal [53,54]. Thus, the Caudal gradient [55] is essentially the inverse of the Bicoid gradient, and Caudal functions as a transcription activator in the posterior of the egg, further influencing expression of the gap gene network. Some of the important interactions in the gap gene network are shown in Figure 1A, although it should be noted that this overview is an oversimplification and does not consider differences in maternal and zygotic factor expression over time, nor does it consider all of the factors involved.

From the outset, we chose to model our system around elements of the *Drosophila* gap gene network (Figure 1A), because we could study many elements of morphogenesis, such as gradient formation and the sufficiency of cross-repression for setting pattern boundaries, without the need for considering multiple cells, membrane-bound receptors, and cell-to-cell interactions. Using an in vitro model, we wanted to address the following questions about patterning. First, how easy is it to generate an expression pattern in a gradient, using a diffusing activator from a localised source? Second, one of the outstanding issues in the field is to what extent correct positioning of the gap protein domain boundaries is specified by maternal morphogen gradients and by cross-repression between gap genes; a recent model suggests that repression is crucial for patterning and that threshold-dependent interpretation of the maternal morphogen concentration is not sufficient [56]. We therefore wanted to test the effect of transcription repression on pattern formation directly, by progressively adding more repression interactions in a designed gene network. Third, uniform degradation of a diffusing morphogen is often assumed to account for steady-state gradient formation, so we set out to test the effects of adding controlled degradation to an in vitro patterning system. Finally, we wanted to use our model to see how the scale and pattern of the system are affected by the relative rates of diffusion of individual components, and whether nonuniform diffusion of activators and inhibitors are required to form a pattern.

## Results

### Design of the Network and Development of the In Vitro Experimental Platform

We began by designing a simplified gene network to emulate elements of the *Drosophila* gap gene system (Figure 1). The aim was to develop a fully synthetic approach in which protein analogues completely unrelated to *Drosophila* would emulate some of the transcription activation and repression interactions thought to be important for patterning in the gap gene system. As activators, two sequence-specific polymerases were employed, T7 and SP6, that have been used successfully by others to engineer gene networks [57]. These two polymerases bind to their respective consensus DNA recognition sites to initiate transcription, and thus represent an extremely simplified mode of transcription when compared to the multifactor complexes required for eukaryotic transcription (reviewed in [6]).

T7 polymerase was chosen to be the “master activator” of the system and, by crude analogy, was expected to carry out some of the functions of Bicoid, namely transcription activation of downstream members in the gap gene hierarchy, in a concentration-dependent manner, from a localised source [10,11]. In *Drosophila*, the Bicoid morphogen gradient initially controls the shape of the Caudal protein gradient through translational repression of maternal mRNA [53,54], although later Caudal expression is under zygotic transcriptional control. To simplify this level of complexity, we decided to model Caudal transcription activation by a second gradient of T7 polymerase, from the opposite pole to our primary “Bicoid” gradient (compare Figure 1A and 1C). Residual activation between other members of the gap gene members (e.g., Figure 1A, Hunchback activating Krüppel or Krüppel activating *knirps*) was modelled nonexplicitly by having a homogeneous distribution of a second sequence-specific transcription activator, SP6 polymerase (Figure 1A and 1C).

**Figure 2:**
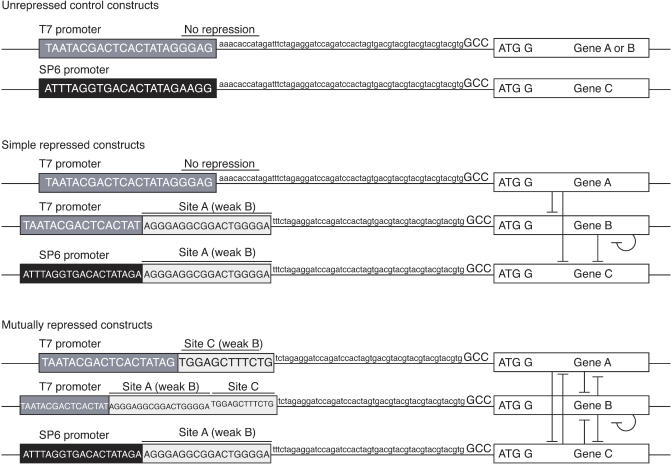
Map of the Constructs Used in This Study The repressor binding sites overlap with T7 or SP6 promoters and vary between constructs. In this way, it is possible to alter the connectivity of the repressive interactions by the products of genes A, B, and C. Repressive interactions are denoted by T-bars. The start codon of each gene is in Kozak context and is denoted by “GCC ATG G.”

Repression interactions between gap gene members were modelled by constructing three site-specific repressors to represent the repressor activities of Hunchback, Giant, and Krüppel (Figure 1B, repressors A, B, and C, respectively). The repressors were derived from artificial zinc finger DNA-binding domains that were engineered by phage display [58]. Variable gene-repression networks were therefore constructed by placing binding sites for the repressors in the appropriate gene expression constructs (see for comparison Figure 2). Repressor sites were either overlapping with the polymerase initiation sites (demonstrated to be effective using triplex-forming oligonucleotides [59]), or immediately downstream of the initiation sites (Figure 2). Therefore, by changing the identity of the repressor sites, the connectivity of the network could readily be modified to add or remove cross-repressive interactions.

Key to the strategy was the development of an experimental platform in which to model the volume of the *Drosophila* embryo and to carry out artificial gene network reactions. Plastic chambers were therefore developed, constructed over printed templates on petri dishes (Figure 1D; see also Materials and Methods and Protocol S1). The chambers were filled with a customised transcription-translation mixture, allowing gene network reactions to be carried out in situ. Additionally, small bar magnets were fixed under the chamber to create a spatially defined array, over which paramagnetic beads could be dispensed. By coating such streptavidin-linked beads with biotinylated PCR products, specific gene network constructs were tethered and sublocalised on the array.

Furthermore, ultra-low melting point agarose was added to the transcription-translation mixture, both to increase viscosity and to allow the reaction to be “fixed” in a gelling step at 4 °C. Through fixing, gel slices could be excised and assayed by Western blotting against FLAG-epitope tags on the expressed proteins. This design therefore enabled quantification of each output species present in the network (genes A, B, and C) for any given chamber position and time point.

The chambers were constructed such that the system components could be pipetted wherever desired, either homogeneously mixed with the transcription-translation mix or pipetted at defined loci, such as at the edges or “poles” of the chamber. As described above, these system components included the two soluble, purified transcription activators (T7 and SP6 polymerases) and three bead-tethered zinc finger transcription-repressor constructs, A, B, and C, which were themselves activated by the polymerases and could cross-repress each other (see Figure 1).

Positional information was therefore introduced into the artificial system in two ways. First, by injecting purified T7 polymerase at either pole of the chamber, the Bicoid activator distribution could be transiently modelled. Second, beads coated with different gene network constructs (genes A, B, and C), could be fixed at different positions on the magnetic array (see Figure 1D).

For example, repressor A genes were placed solely at the chamber edges (“poles”) to model, loosely, the distribution of embryonic Hunchback activity. This part of the model is a significant oversimplification: Although Hunchback is eventually expressed in two domains, one anterior and one posterior [48], it is only expressed anteriorly in the early embryo. Furthermore, while maternal *hunchback* mRNA is evenly distributed, the anterior domain of Hunchback protein forms through zygotic translation and transcription activation (under the control of Bicoid), while maternal RNA is translationally repressed posteriorly, under the influence of Nanos [49,60,61]. In the later phase of *hunchback* regulation, the posterior Hunchback domain forms through a combination of factors, including activation by Tailless [62,63] and *hunchback* autoactivation [64].

To complete the model, the genes for repressors B and C were distributed uniformly throughout the chamber on magnetic beads, to represent the ubiquitous distribution of genes in nuclei, throughout the embryo. Therefore, the spatial expression of genes B and C, who represent the downstream gap gene members *giant* and *Krüppel* (see Figure 1A and 1C), was dependent on differential activation by the T7 polymerase gradient and crossregulation between gene network members. However, it should be noted that the initial expression of these genes in *Drosophila* may not be achieved by crossregulation, because localised mRNA is seen before any protein is detectable (Krüppel and Giant are only detected unambiguously in early cycle 13 [46]).

### Pattern Generation In Vitro from a Transcription Network

In our first experiments, we constructed a simple, minimal network with sequential transcription activation and repression (see Figure 1B and 1C). Although this basic system is far less complex than the *Drosophila* gap gene system, it was indeed sufficient to generate a crude target behaviour (see Figure 1E and 1F). Qualitatively, the pattern can be explained as follows, Gene A is activated by T7 polymerase from its source at either end of the chamber, and so is expressed most highly at these poles. Gene B is similarly activated, and so it is also less expressed in the middle of the cell. However, since gene B is repressed by protein A, its levels are also reduced at either pole. Finally, Gene C is activated by a ubiquitous SP6 polymerase, but is repressed by proteins A and B, and is consequently centrally distributed.

**Figure 3:**
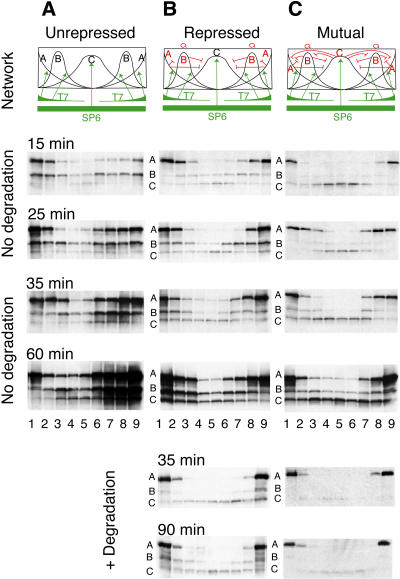
Alternative Gene Networks At five set time points (15, 25, 35, 60, and 90 min), transcription-translation chambers were dissected into nine slabs for Western blot analysis. (A) Control network with no repression sites between genes A, B, and C. (B) Minimally repressed network (compare Figure 1). (C) Mutual repression network with extensive negative interactions between species. Adding protease (“+ Degradation”) creates weak but time-stable patterns for both the “Repressed” and “Mutual” networks (35 versus 90 min). Quantitated graphs for the above data are available in Protocol S1.

Progressing from the minimal network, we explored systems with a variety of connectivities (Figure 3), including a control network without repression interactions (Figure 3A), and one with extensive mutual or feedback interactions (Figure 3C). These were compared with the original network (Figure 3B) in a series of time-course experiments. Generally, we observed that the more repression interactions in a system, the lower the overall protein production but the “sharper” the pattern.

All patterns degenerated to a significant degree by 60 min, indicating the transience of the system (Figure 3, 60 min). However, by adding Factor Xa protease, we were able approximately to match levels of production and degradation. Thus, the outputs became sharper, weaker, and more dynamically stable, hardly varying between 35 and 90 min (Figure 3B and 3C, “+ Degradation”).

### Computer Exploration of Parameter Space

**Figure 4:**
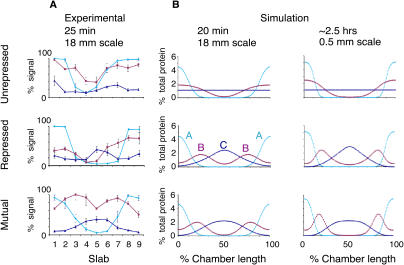
Comparison of Experimental Data and Computer Simulations Data are shown for the three gene networks described in Figure 3, showing outputs for proteins A (cyan), B (magenta) and C (dark blue). (A) Quantitated Western blot data from Figure 3, after 25 min. (B) Simulation data plotted as percentage of total output protein against chamber length, at the chamber (18-mm) or *Drosophila* (0.5-mm) scale. The model is described in full in Protocol S1.

To study parameter sensitivity in our system more comprehensively, we constructed a computer model of the chamber and networks (Protocol S1). A series of coupled differential equations were simulated, yielding expression levels of gene products A, B, and C, for the three different levels of network connectivity coded by our gene network designs (Figure 4). The modelling was carried out at two scales, that of our experimental system (18 mm long) and that of a *Drosophila* embryo (500 μm long). As in the experimental system (Figures 3 and 4A), the simulations revealed a large difference of pattern between the unrepressed and repressed systems. The patterns are more similar, however, between the simple and mutually repressed networks (Figure 4B) but, as in our in vitro experiments (e.g., see Figure 3B and 3C, 15 min), adding feedback repression makes the peaks better resolved.

**Figure 5:**
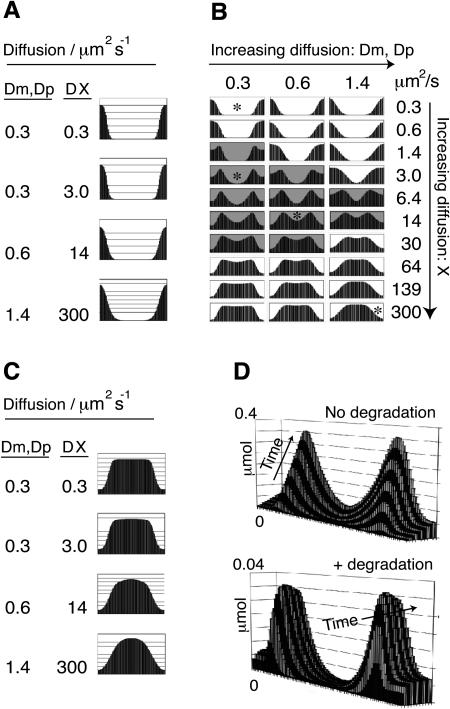
Varying Diffusion and Degradation Parameters Computer model of gene network, scaled to *Drosophila* length (0.5 mm). Diffusion parameters are varied for mRNA (Dm), protein (Dp), and T7 activator (DX). Data are plotted as percentage of total output protein (y-axes) against chamber position (x-axes), for 10-min simulations. (A) Outputs for protein A. (B) Output for protein B. Graphs with “target behaviour” are shaded grey, and the four asterisks mark the parameter sets used to generate outputs for proteins A and C. (C) Outputs for protein C. (D) Effect of adding protease degradation to B-output, shown at 15-min intervals, over a 2.5-h time course (parameters: DX = 0.43 μm^2^s^−1^; Dm = Dp = 0.02 μm^2^s^−1^; t_1/2_ = 770 s).

Next, we explored the sensitivity of the simple repression network to diffusion parameters (Figure 5). Generally, we found that the A- and C-peaks were least sensitive to parameter variation, as there are no antagonistic forces against their formation (Figure 5A and 5C). By contrast, gene B is more sensitive: Twin-peak formation correlates with the relative diffusion ratios of activator (T7) and other mRNA/protein components (Figure 5B). To generate “target behaviour,” the activator must diffuse more rapidly than other species, within certain limits (approximately 5- to 50-fold faster for Figure 5B). However, the absolute values (and ratios) for diffusion merely alter the timing of the transient B-peak formation in a system of a given scale. For simplicity, only the 0.5-mm system is illustrated in Figure 5; similar conclusions were drawn from the 18-mm scale model.

As in our chamber experiments, the computer model output became more “time-stable” by adding a degradation element (Figure 5D). *Drosophila* may exploit such mechanisms to some extent, since Bicoid protein degrades in vivo (t_1/2_ ≤ 1800 s [11]), although *bicoid* mRNA is unusually stable [65].

## Discussion

To develop a fully synthetic approach that will emulate elements of gap gene expression domain pattern formation, we created an in vitro transcription-translation system that allows flexible spatial gene network construction. The system is widely applicable, allowing control over factors such as localisation or diffusion, and the ability to add or remove components at will.

### Repressive Interactions and Pattern Formation

A basic aim of our system was to see whether we could engineer a gradient of protein expression, using a diffusing activator from a localised source. We found this task straightforward in the transcription-translation chambers, using injected T7 polymerase, and this led us to try more complex expression-repression interactions. We constructed three types of gene network—unrepressed, simple repressed, and mutually repressed (see Figure 3)—representing different levels of network connectivity. Generally, in both our in vitro and computer models, we found that adding more connections resulted in better-resolved patterning, although the absolute levels of gene expression were reduced. Our in vitro results are essentially qualitative at this stage, but appear to agree with the observations of others—that crossrepression is crucial for the control of patterning boundaries [56]. It will be interesting to learn whether more sophisticated elements can be engineered into the system to begin to emulate the more complex features of gap gene expression domain patterning. For example, dynamic anterior shifts are seen in domain expression over time because of asymmetric gap-gap crossrepression [56]. Asymmetric repression and other circuits could, in the future, be engineered into our chambers by altering the repressor binding sites in the appropriate constructs. Such a system would require component turnover to achieve steady-state patterning. We have begun to tackle this project through our experiments with controlled protease degradation, but a further requirement would be to have autocatalytic production of T7 polymerase from a localised source, rather than the injected pulse of purified polymerase in our current model.

Interestingly, our experimental data showed a reproducible degree of patterning even in the unrepressed system (Figure 3A, 15 min, C output). Because gene C is activated by a separate polymerase (SP6), this patterning cannot result from competition for activator. Therefore, competition for other resources (such as ribosomes, nucleotides, and tRNAs) may allow A and B to “inhibit” C. Indeed, supplying extra components (particularly wheat germ extract and SP6 polymerase together) increases protein production under these conditions, including that of C (unpublished data). If competition can generate patterns, albeit less well defined ones than repression-connected networks, this could perhaps represent an evolutionary “network precursor” state: Weak patterns could be generated by localisation and competition between factors, and these could later be consolidated by evolution of a “true” negative network connection. However, this hypothesis may not be relevant to the situation inside an insect egg, as this has probably evolved to deliver nutrients very efficiently to the embryo, even at a very primitive evolutionary stage. It therefore remains to be seen whether competition effects would be as significant in vivo.

Since our models use minimal components to achieve spatial pattern formation, they demonstrate the ease with which very simple networks might evolve. Patterning may be achieved with only localisation, diffusion, and some kind of functional network connection, such as transcription activation, competition, or repression. The addition of extra layers of network properties, such as controlled degradation, could then fix and stabilize such patterns. In fact, since sublocalisation—followed by stepwise addition of network components—is sufficient to generate crude patterns, it might provide a plausible mechanism for early spatial network evolution inside a single cell. However, it should be noted that gap gene expression domain patterning probably evolved by a different mechanism, from an earlier multicellular state, where segments were added sequentially through polar growth. In fact, *bicoid* is absent in most other insects, and it has been proposed that *Drosophila* evolved *bicoid* by duplication of the homeodomain-encoding gene *zerknüllt,* found in lower Diptera [66].

### Diffusion Rates and Patterning

We were intrigued by our observations, both in vitro and in silico, that patterning required the activator molecule to diffuse or propagate more rapidly than the inhibitors. This is interesting because it is the opposite of the M-G system (described in the Introduction and reviewed in [12]). Long-range activation is not unknown in chemical patterning systems [67], although many biological models appear to require the M-G criterion for long-range inhibition (e.g., [68]). The other obvious differences between our system and the M-G model are the initial localisation of components and the lack of autocatalysis of the activator. It will be interesting to determine whether such M-G patterning systems can be recreated in our chambers, once further factors are considered, such as the avoidance of “autocatalytic explosions” or “global inhibitions.”

The computer model that we developed allowed us to test a broad range of parameters, such as diffusion and degradation rates (Figure 5), revealing differences between the requirements for patterning among the different species in the gene network. First, the more-connected member of the network (gene B) was much more sensitive to parameter variation than the less-connected members (genes A and C). This is perhaps to be expected, since protein B has two separate boundaries of expression (defined as a function of T7 distribution and both A and C expression), whereas proteins A and C have only single “edges” to be defined.

Another important feature of the system emerged when scaling the parameters for the model patterns to *Drosophila* scale (Figure 5, 0.5 mm “embryo,” 2.5 h). We found that, assuming simple diffusion, B-peak formation was compatible only with unphysiologically slow diffusion values (diffusion constants for mRNA [Dm] and protein [Dp] = 0.02 μm^2^s^−1^; for T7 activator [DX] = 0.43 μm^2^s^−1^). Since cellular proteins are expected to diffuse more rapidly (approximately 1–100 μm^2^s^−1^), this could be an artefact, reflecting the simplicity of our model. Nonetheless, simple diffusion still appears too rapid to account for *Drosophila*-scale patterning. It should be noted that a potential barrier to free diffusion is the active nuclear import of Bicoid and Hunchback [69]. In a separate example, diffusion of pair-rule transcripts is overridden by microtubule transport [70]. Controlled sublocalisation may therefore be crucial to limit apparent diffusion in vivo, allowing more precise patterning.

### Perspectives

The understanding of how precision of patterning is achieved in *Drosophila* is still far from complete. In a recent study, it was shown that the Bicoid profile is far more variable between embryos than that of Hunchback, but the mechanism by which this noise is filtered remains unknown [71]. As more and more detailed experimental data are collected [72], and new mechanisms are proposed to account for patterning, it will be important to test the sufficiency of these mechanisms through experimental reconstitution. For such purposes, the chambers described here may be easily adapted to test different hypotheses. In vitro systems are a useful first step towards testing the sufficiency of a network—which might then be reengineered in the original target organism.

Combining simple reconstruction with theoretical modelling is a useful tool to discover and test general design principles in gene networks [73,74,75,76]. Until now, however, the spatial component essential in many biological processes has been ignored in these approaches. We anticipate that other networks, such as signalling cascades or metabolic networks, might also be studied using our system and that the spatial element, introduced through the beads, might provide new insights into complex systems.

## Materials and Methods

### 

#### Magnetic chamber construction

A detailed, step-by-step description of the construction of the chamber can be found in Protocol S1. Briefly, nine stirring-bar magnets (1.5 mm × 8 mm; VWR International, Vienna, Austria; #4429025) were inserted vertically into a plasticine-filled standard petri dish, creating a magnetic array (see Figure 1D). Construction was guided with a grid template, laser-printed on a transparent acetate sheet, and fixed over the magnets and plasticine. The template was a 3 mm × 18 rectangle with nine subdivisions (“slabs”). A sterile cell culture dish (Nalge Nunc, Rochester, New York, United States; #150350) was fixed immediately above the magnetic array. Chamber borders (1 mm deep) were constructed on the base of this second dish, following the template, using strips cut from adhesive Hybriwell chambers (Sigma-Aldrich, St. Louis, Missouri, United States; #H1159–100EA).

#### Gene network constructs

Maps of the constructs are illustrated in Figure 2. Repressors A, B, and C were derived from previously engineered zinc fingers [58]. Repressor A contained six zinc fingers, recognising the sequence 5′- AGGGAGGCGGACTGGGGA-3′, fused to the residues 11–55 of the Kox-1 repression domain [77] and a six-repeat FLAG epitope tag [78]. Repressor B contained six zinc fingers, recognising the sequence 5′- AGGGAGGCGGGAGCTTTC-3′ and fused to a three-repeat FLAG-tag. Repressor C contained three zinc fingers, recognising the sequence 5′- GGAGCTTTC-3′, fused to the Kox domain and a three-repeat FLAG-tag. The following polymerase consensus promoter regions were used: T7, 5′- TAATACGACTCACTATA**G** GGAG-3′; SP6, 5′- ATTTAGGTGACACTATA**G** AAGGG-3′. The gene network promoters were linked with neutral or repressor sites to the polymerase promoters.

In the following nucleotide sequences, zinc finger binding sites are indicated in lowercase, initiation nucleotides in bold, and promoter overlaps underlined. Unrepressed T7, 5′- TAATACGACTCACTATA**G** GGAGAAACACCATAG-3′ (see Figure 3A, constructs A and B, and Figure 3B, construct A). Unrepressed SP6, 5′- ATTTAGGTGACACTATA**G** AAGGGAAACACCATAG-3′ (see Figure 3A, construct C). T7 repressed by A (and weakly by B), 5′- TAATACGACTCACTATa**g**ggaggcggactgggga -3′ (see Figure 3B, construct B). SP6 repressed by A (and weakly by B), 5′- ATTTAGGTGACACTATA**G**Aagggaggcggactgggga-3′ (see Figure 3B and 3C, construct C). T7 repressed by A and C (and weakly by B), 5′- TAATACGACTCACTATa**g**ggaggcggactggggaTggagctttc-3′ (see Figure 3C, construct B). T7 repressed by C (and weakly by B), 5′- TAATACGACTCACTATA**G**ggagctttc-3′ (see Figure 3C, construct A). Constructs were cloned in pCaSpeR4, sequenced, and used to generate PCR DNA for in vitro transcription-translation.

#### Gene network reactions

Paramagnetic beads were coated with PCR DNA (with one primer biotinylated) using a Dynabeads Kilobase Binder Kit (Dynal, Oslo, Norway; #601.01). Typically, gene A was used at 800 fmol per 10 μl of beads, resuspended in 8 μl of water; 200 fmol of gene B and 140 fmol of gene C were combined with 20 μl of beads, and resuspended in 20 μl of water.

Transcription-translation mixture was prepared that included 2.5 μl of water; 28 μl of ultra-low melting point agarose (Sigma; #A2576) solution (prepared as 1.5% [w/v] in boiling water and cooled to 30 °C); and TNT Coupled Wheat Germ Extract System (Promega, Madison, Wisconsin, United States; #L4130 and #L4140), which comprised 20 μl of TNT wheat germ extract, 1.2 μl of TNT reaction buffer, 0.6 μl of amino acid mixture (1 mM), 1.2 μl of RNasin (not included in TNT kit), and 0.5 μl of SP6 polymerase. 54 μl of this mixture was dispensed per chamber.

For degradation experiments, 2.25 units of Factor Xa (Amersham Biosciences, Little Chalfont, United Kingdom) were added per chamber. Coated Dynabeads were injected at appropriate positions over the magnetic array: typically, 100 fmol of gene A (1 μl), 5 fmol of gene B, and 3.5 fmol of gene C (0.5 μl). T7 polymerase (0.5 μl; from Promega TNT kit) was immediately injected at the chamber edges. After timed incubations at 25 °C, chambers were transferred to 4 °C for 35 min, to form a gel. Gel slices were cut with a razor blade (guided by the printed template) and aspirated with a P10 Gilson pipette. Samples were mixed with 10 μl of SDS-loading buffer and analysed by SDS-PAGE, Western blotting, and ECL, with anti-M2 FLAG antibody (Sigma; #F3165). Further details on this step can be found in Protocol S1.

#### Computer modelling

A Perl script was written to simulate the diffusion-coupled expression of genes A, B, and C, by T7 and SP6 phage polymerases, in a translation extract. The program parameters and script are fully described in Protocols S1–S3. 18 mm-scale chamber model: Parameters included separate diffusion (and degradation) rates for RNA and protein; a separate apparent diffusion for injected T7, modelled from experimental observations (rapid initial diffusion with exponential decay; Section 5 of Protocol S1); estimated binding constants for all interacting species (zinc finger dissociation constants were estimated from previous work on related three- and six-finger constructs [58,79,80]); and estimated transcription-translation rates. For adapting the model to the 0.5-mm *Drosophila* scale, chamber size was scaled down, and only simple diffusion was allowed for all components; for simplicity, transcription-translation rates were not varied (Section 3 of Protocol S1)*.*

## Supporting Information

Protocol S1 Detailed Description of Model (1.2 MB PDF).

Protocol S2 Parameter File for Simulations This file contains the default parameters for the computer model in a format that can be read by the Perl script.
(5 KB DOC).

Protocol S3 Computer Program Script for Simulations This text file is a Perl script to run the computer simulations described in the manuscript.
(22 KB DOC).

### Accession Numbers

The Locuslink (http://www.ncbi.nlm.nih.gov/LocusLink/, or GeneID (www.ncbi.nlm.nih.gov/entrez/query.fcgi?db=gene, accession numbers of the genes and proteins discussed in this paper are Bicoid (40830), *caudal* (35341), *giant* (31227), *huckebein* (40549), *hunchback* (41032), *knirps* (40287), *Krüppel* (38012)*,* Nanos (42297), *tailless* (43656), Torso (35717), and *zerknüllt* (40828).

## Abbreviations

Dm: diffusion constant for mRNA
Dp: diffusion constant for protein
DX: diffusion constant for T7 activator
M-G: Meinhardt-Gierer
