# Supplementary material for: Engineering Gene Networks to Emulate Drosophila Embryonic Pattern Formation
Source: PLoS Biol. 2005 Feb 22;3(3):e64. doi: 10.1371/journal.pbio.0030064 (PMC1044831; doi:10.1371/journal.pbio.0030064)
Supplement: Supplementary file 1 [file pbio.0030064.sd001.pdf]

Supporting Information:

Detailed Description of Model: Engineering Gene Networks to Emulate Drosophila Embryonic Pattern Formation.

Mark Isalan, Caroline Lemerle & Luis Serrano

EMBL, Structures & Biocomputing, Meyerhofstrasse 1, Heidelberg D-69117, Germany

Summary

This section describes the model and parameters used to generate the data in Figures 3 and 4 of the main text. The model simulates a gene network comprising repressor proteins, termed A, B and C. These generate a transient stripe pattern under the influence of factors such as gene localisation, transcription-activation/repression, translation, diffusion and degradation. As well as describing the model, we provide the Perl script that we used to simulate the network. The simulation script is included as *a* separate file *(*text file: simul.pl) from which it can be run using any machine with Perl installed. We also provide the parameter sets (param.txt) used for generating data for Fig. 4 in the main paper, together with instructions on how to deploy them.

In Appendix A, quantitated data are provided for the Western blots in Figure 2 of the main text. In Appendix B, step-by-step instructions are given on how to build a transcription-translation chamber.

Contents

Summary 1

Implementation
1. General system description 3
2. Spatio-temporal resolution of the system 3
3. System parameters 3
   Table S1: System parameters: default values 4
4. Simulation equations 5
   Table S2: Difference equations for each species 5
5. Diffusion at boundaries and T7 diffusion 6
   Fig. S1: Horizontal diffusion 8
   Fig. S2: Vertical diffusion. 9
6. Installing perl and running the script 10
7. Choice of network model 10
8. Loading parameter values 11
   Table S3: Sample parameter sets 11
9. Output options: contents and location 12

Appendix A
Quantitated Western Blot data 14
   Fig. S3: Quantitated data for Fig 2 in the main text 14
Appendix B
Step-by-step construction of a transcription-translation chamber reaction . 15

Implementation

1. *General system description*

The gene network consists of X and Y activator proteins (representing T7 and SP6 in the experimental system), and 3 target genes coding for repressor proteins A, B and C. The time-dependent distribution of all activator proteins and gene products is simulated using a system of difference equations, one for each relevant species (ie all mobile species with a heterogeneous spatial distribution as found in column 2 of Table S1).

2. *Spatio-temporal resolution of the system*

For the simulation of the experimental system, each of the 9 slabs composing the 'egg' chamber was divided into a fixed number of minislabs (typically 10) to get a higher spatial resolution of the peaks of protein forming as the system evolves, compared to that of the experimental data (1 per slab; see Fig 1 in main text).

The temporal resolution was always chosen to avoid numerical instabilities that are likely to arise when the following inequality does not hold : $\Delta t<4*\Delta z^2/D$, where D is the effective diffusion constant for the species considered, Δt is the timestep, and Δz is the unit space (Euler's method; *Institutionum Calculi Integralis,* 1768)

3. *System parameters*

System parameters include initial spatial distributions of species, first-order rates for transcription, translation and degradation processes, matrix of binding constants for transcription factor-promoter complexes. For a complete description of parameters including default values for the repressed network, see Table S1.

We determined the binding affinities of the 3-finger DNA-binding subdomains of the repressors (proteins A, B and C), both by ELISA and gel retardation assays (Nat Biotechnol. 2001 Jul;19(7):656-60). For example, the 3-finger DNA-binding domain of Repressor C (previously called HIV-F in the Nature Biotech. paper) has an apparent Kd = 13 nM (Affinity = 77 uM-1). This value is used in the model. However, we merely estimated the binding affinities of the two six-finger fusions, Repressors A and B, based on the affinities of their component 3-finger domains and on our previous experience in making 6-finger fusions (Proc Natl Acad Sci U S A. 2001 Feb 13;98(4):1437-41.; Proc Natl Acad Sci U S A. 2001 Feb 13;98(4):1432-6 and unpublished results).

Repressor A is a fusion two 3-finger proteins with Kd's determined to be around 1 nM each (previously called HIV-A and B, Nat Biotechnol. 2001 Jul;19(7):656-60). The six-finger binding constant is estimated to be: Kd = 0.1 nM (Affinity = 1e4 uM-1), based on comparisons with the very similar protein "TF(1–3)-flex-ZIF" (about 60 pM; Proc Natl Acad Sci U S A. 2001 Feb 13;98(4):1432-6). Repressor B is a fusion two 3-finger proteins with individual Kd's of ~1 nM and 13nM(ELISA)/20nM(BANDSHIFT) respectively (previously called HIV-A and F, Nat Biotechnol. 2001 Jul;19(7):656-60). The six-finger binding constant is conservatively estimated to be: Kd = 2 nM (Affinity = 5e2 uM-1).

Table S1:

System parameters: default values for the mutually repressed network

Parameter type	Parameter name	Default value	Unit	Description	Comments
Spatial parameters	slablength	2000	Um	length of gel slab	the complete chamber is divided into 9 such slabs
	minislablength	200	"	length of unit space	defines spatial resolution
	slabvol	6e-6	L	volume of gel slab	=2mm*3mm*1mm
Initial amounts	dnaA	1e-7	umol	amount of gene A in slabs 1 and 9	other slabs contain no gene A
	dnaB	5e-9	"	amount of gene B in each slab	
	dnaC	3.5e-9	"	amount of gene C in each slab	
	X0	1e-6	"	amount of protein T7 in slabs 1 and 9	other slabs contain no T7
	Y0	1e-6	"	amount of protein SP6 in each slab	
Binding constants	KassX	2.5e2	l.uM-1	association constant of T7 with the T7 promoter	T7 promoter drives genes A and B cf JMB 273 775
	KassY	2.5e2	"	association constant of SP6 with the SP6 promoter	SP6 promoter drives gene C
	KassAp	1e4	"	association constant of protein A with its target dna sequence	target dna for this protein is found upstream of genes B and C
	KassBp	5e2	"	association constant of protein B with its target dna sequence	target dna for this protein is found upstream of genes A,B and C
	KassCp	77	"	association constant of protein C with its target dna sequence	target dna for this protein is found upstream of genes A and B
Process rates	ktr	1e-2	s-1	transcription rate from T7 or SP6-bound promoters	Ref. biochem vol26 p2690-
	ktl	1e-2	"	translation rate	Ref: embo j vol18 p6705 .3mn-1, ie 30x less than in vivo
	kdegr	0	"	degradation constant for mRNAs	Kit assay : less than 1% of RNA degraded in 1 hour with 20u of extract
	kdegp	0	"	degradation constant for proteins	
Diffusion	kX	1e-3	"	exponential decay constant	let kX=0 for simple diffusion
	DX0	25000	um^2.s-1	initial apparent diffusion constant for T7 protein	Accelerated diffusion is modelled to compensate for effects generated upon deposit of T7 at start of experiment
	Dres	80	"	residual diffusion constant for T7	"
	Dm	1000	"	effective diffusion constant for RNAs	
	Dp	1000	"	effective diffusion constant for proteins	Tuned to see near-constant level of T7 in all slabs after 2 hours (data not shown)
Temporal parameters	timestep	1	s	unit timestep for simulations	
	runtime	3600	"	simulation time at end of run	
Output options	sampleperiod	300	"	periodicity of outputs	
	outputs	(0,0,0,1, 0,1,0,1)	boolean list	list specifying the species for which an output should be generated	complete list is (X,Y,Am,Ap,Bm,Bp,Cm,Cp)

N.b : X and Y designate T7 and SP6 activator proteins. m and p subscripts stand for mRNA and protein respectively

4. *Simulation equations*

For each species E, the dynamics of its concentration at time t and position s are governed by the following classical difference equation:

(1) $\Delta E = E(s,t+1) - E(s,t) = \Delta t * (diff + prod - deg)$

where the terms diff, prod and deg (accounting respectively for diffusion, production and degradation of each species) depend on the behaviour of the species. These are shown explicitly in Table S2, below.

Table S2:

Difference equations for each species in the repressed network

**Species**	**diff#**	**prod**	**deg**
X	DX/Δz^2*[E(s+1,t)+E(s-1,t)-2*E(s,t)]	0	kdegp*E(s,t)
Y	Dp/Δz^2*[E(s+1,t)+E(s-1,t)-2*E(s,t)]	0	"
Am	Dm/Δz^2*[E(s+1,t)+E(s-1,t)-2*E(s,t)]	ktr*dnaA(s)/[1+KX/X(s,t)]	kdegr*E(s,t)
Bm	"	Ktr*dnaB(s)*X(s,t)/KX/[1+X(s,t)/KX+Ap(s,t)/KA+Bp(s,t)/KB]	"
Cm	"	Ktr*dnaC(s)*X(s,t)/KX/[1+X(s,t)/KX+Ap(s,t)/KA+Bp(s,t)/KB]	"
Ap	Dp/Δz^2*[E(s+1,t)+E(s-1,t)-2*E(s,t)]	ktl*Am(s,t)	kdegp*E(s,t)
Bp	"	ktl*Bm(s,t)	"
Cp	"	ktl*Cm(s,t)	"

terms valid as long as s corresponds to an inner position. For boundary positions, see Section 5.

5. *Diffusion at boundaries and T7 diffusion*

At the two boundary positions of the system (first and last minislabs), the diffusion term diff, from equation 1, is modified to account for the mobility constraint imposed on the species by the presence of a wall at each end.

Thus, the diffusion factor E(s+1,t)+E(s-1,t)-2*E(s,t) used for all inner slabs (see column 1 of Table 2) is replaced by E(s+1,t)-E(s,t) for the left end position (as defined by s=0), and E(s-1,t)-E(s,t) for the right end position (as defined by the $slablength parameter). Note that this applies for all species in the system.

In adjusting the model to the experimental system and accounting for the fact that T7 polymerase is the only component added dynamically to a system that has settled, we adopted an empirical approach to modelling "flow' of T7, based on our experimental observations for apparent diffusion (see Figs. S1, S2, below).

As shown in equation 2, we substituted the constant diffusion term of T7 for an exponentially-decreasing apparent diffusion factor DX, dropping from a very high initial value (DX0) to a physiological residual value (Dres).

(2) $$DX = (DX0 - Dres) * \exp(- kX * t) + Dres$$

Note: that to recover a simple diffusion model for T7 from equation 2 (as for all other components) then let kX=0 (Dres can be set to any value, as it cancels out from the equation).

The basis for this approach arose from our initial observations that the T7 gradient formation appeared too rapid to be explained by diffusion, although the distribution was reproducible (indirectly observed by protein expression in our Western blots). Therefore, to investigate this phenomenon, we carried out a series of diffusion experiments in replica chamber conditions (containing agarose / in vitro translation mix / etc.) but replacing the indirectly-observed T7 pol. activity with a directly-observed coloured species; either of bromophenol blue (~0.7 kD) or the red protein, cytochrome C (12.5 kD); note that T7 polymerase itself is 99 kD. 1 µl of saturated aqueous solution of either coloured species was injected at the chamber edge and 'diffusion' was allowed to occur (Fig S1A).

Snapshots of the cuvettes were taken at regular time intervals using a scanner, producing images in .tif format which were processed with Matlab. The images were converted into matrices of unsigned 8-bit integers (values from 0 to 255) and after collapsing on one dimension, subtracting background and normalizing to peak values, the images gave the profiles shown in Fig S1B.

Freely diffusing molecules moving away from the water/dense coloured solution interface are expected to yield a spreading Gaussian distribution of concentration values, where the following ratio: $x^2/[\log(1/cnorm)*t]$ provides an estimate of the diffusion constant value. This reads as the slope of the linear regression on the data points with x-coordinates [log(1/cnorm)*t] and y-coordinates x^2. Fitting our experimental profiles to simple Gaussians yielded an 'apparent diffusion constant' for

each time point of our data. These values were far too high for simple diffusion (completely unphysical), but could be conveniently described by 2 parameters: an initial ‘apparent diffusion’ constant DX0 around 2.5e4 μm^2/s, and an exponential decay constant k of 1e-3 s^{-1}, describing the slowing-down of injected species over time (Fig S1 C,D,E).

In our modelling of the experimental system, we introduced a third parameter: the residual 'apparent diffusion constant.' This was given the approximate physiological value of 80 μm^2/s to account for the mobility of the injected species after settling of the system (when simple diffusion becomes a dominant form of mobility).

A question remains, namely what possible mechanism could account for the rapid, decaying ‘diffusion’ patterns observed? We suspected that gravity/density-differences provided at least some of the driving force to the injected species: all the species tested (including T7) appear to be denser than the chamber medium. Therefore, after injection, it appears that the species sink and 'spread sideways' along the plastic chamber floor. To test this assertion, we carried out further diffusion experiments in a modified vertical chamber, where the coloured species were injected at the bottom and only allowed to diffuse against gravity (limited through the chamber boundaries; see Fig. S2).

In contrast to the horizontal situation, the vertical experiment obtained slower ‘apparent diffusion’ constants (in the order of 5e3 μm^2/s), although these were still too rapid for expected values for diffusion in water (self-diffusion constant of water molecules in water: ~2e3 μm^2/s[1]). One possible cause is the mixing effects of convection currents set up by our desktop scanner (surface temperature ~35˚C; ambient room temperature ~22˚C), although other effects might operate as well.

In summary, we applied the experimentally-derived apparent diffusion function from the horizontal experiments (Supplementary Fig. S1) in our computer model and, on the time/length scale of the artificial chamber, it generates very similar patterns to the simple diffusion that was specified in our *Drosophila*-scale computer model (albeit on different time/length scales; see Fig 3, main text).

[1] David Metzler, *Biochemistry*, 2. Edition, Harcourt-Academic Press (2002)

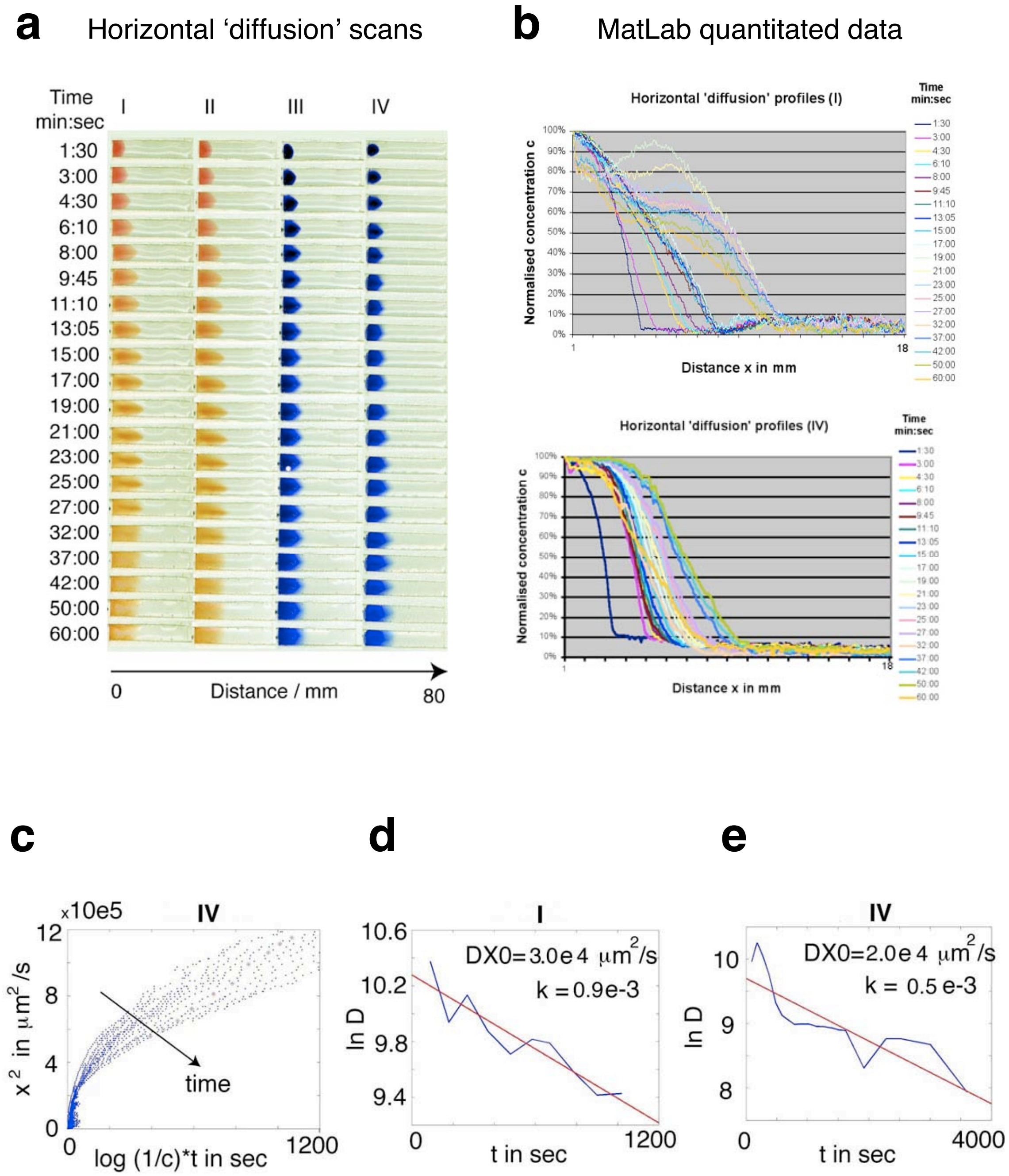

Fig S1 Measurement of 'apparent diffusion' rates of coloured molecules using a flatbed scanner. (a) Time-course showing scans of horizontal diffusion of either the red protein, cytochrome C (replica chambers I,II) or bromophenol blue (replica chambers III, IV). The four 18 mm-length chambers contained standard transcription-translation mix (with 0.75% ultra-low melting point agarose), and were placed flat on a horizontal scanner surface. (b) Scan profile data for chambers I and IV as quantitated by MatLab (similar data were obtained for chambers II and III). (c) Matlab data are plotted as x^2 against [log(1/c)*t]. 'Apparent diffusion' is estimated from the slope of the linear regression for each timepoint. (d,e) Natural logs of apparent diffusion constants D plotted against time. The results show a rapid initial 'flow' that reduces over time, fitting to an exponential decay (for chambers I-IV: DX0 = ~2.5e4 $\mu m^2/s$; k = ~1e-3).

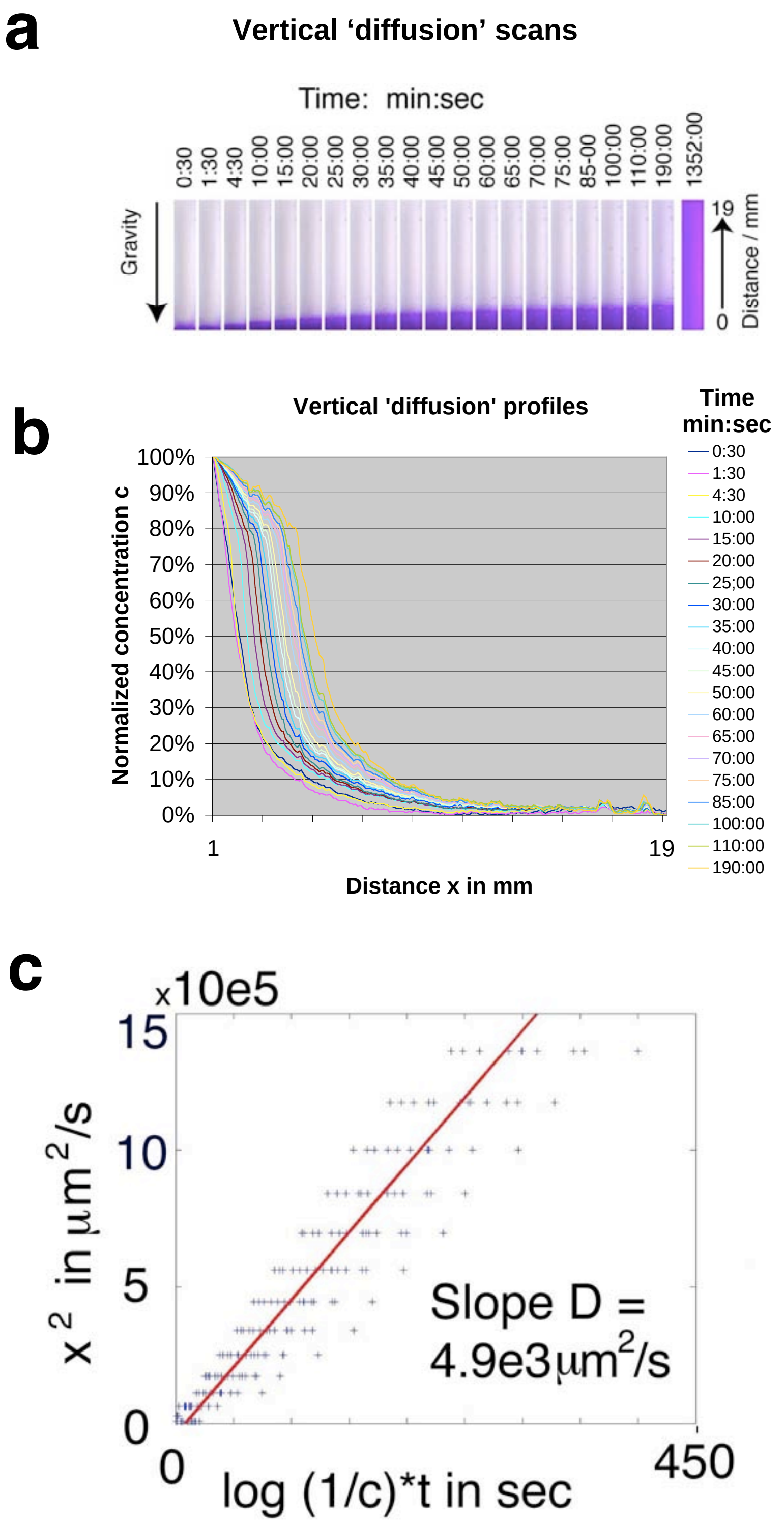

Fig S2 Vertical measurement of ‘apparent diffusion’ rates of coloured molecules using a flatbed scanner. (a) The timecourse experiment was repeated with bromophenol blue in a vertical chamber with gravity acting against the direction of observable ‘apparent diffusion.’ (b) Data were quantitated with MatLab to obtain ‘apparent diffusion’ profiles. (c) Scatter plot: Matlab data are plotted as x^2 against [log(1/c)*t], where the slope of the linear regression, for data points from all vertical time profiles, provides an estimate of the apparent diffusion constant.

6. *Installing Perl and running the script*

Note that Perl must already be installed on the machine for the script to run.

For installing Perl on Windows/Linux/Solaris, download ActivePerl from the following link :
http://www.activestate.com/Products/Download/Get.plex?id=ActivePerl

For installing Perl on Mac OS X-v10.2, see:
http://serverlogistics.com/downloads-jag.php#perl

The simulation script is provided as an accompanying file (*simul.pl)*. The script is annotated, and may be viewed and/or edited using any text editor.

To run, download the file from *Supporting Information* and either double-click the icon of the file or call it from a dos-type window (command prompt) by typing 'perl simul.pl'. To run it with the accompanying '*param.txt'* file also provided, make sure both files are in the same folder.

Before any simulation is carried out, the user is prompted first for the network model to use, the parameter values to be loaded, the output content, finally the destination of output results.

7. *Choice of network model*

As described in the paper, several network connectivities of increasing complexity were tested experimentally. The strength of interaction between network component is specified in the connectivity matrix: a 3 by 3 matrix composed of binding constants where rows correspond to proteins A,B and C while columns correspond to promoters of genes A, B and C (such that, for instance, the matrix element in the first row, second column contains the binding constant between protein A and gene B).

While running the script, the user is first prompted to choose between running a simulation of one of the three pre-defined networks below:

- repressed network
- unrepressed network
- mutually repressed network

and a fourth option, 'custom network' where the user supplies his own connectivity matrix by storing it in a simple tabulated text file (*e.g*. '*mat.txt'*).

8. *Loading parameter values*

Runmodes: ‘d’, ‘m’, ‘p’ or ‘<filename>’

One can run a simulation using the default set of parameter values for the repressed network (see Table S1) by selecting ‘d’ at the prompt, as a test run.

To run a single simulation with a defined set of parameter values, one can choose the manual mode of entry by typing ‘m’, and entering each parameter value individually, when prompted.

Alternatively, one can generate a text file where each line contains a series of tab-delimited parameter values (in the order defined in Table S1), and save it as ‘*param.txt*’ (or any other name) in the same folder as that containing the program script. To run this mode, type either ‘p’, for *param.txt*, or the full name of the file.

Table S3:

Sample parameter sets

Reference		# run	slablength	[illegible]	slabvol	dnaA	dnaB	dnaC	X0	Y0	KassX	KassY	KassAp	KassBp	ktr	ktl	kdegr	kdegp	kX	DX0	Dres	Dm	Dp	timestep	run-time	sampleper
units			um	um	ul	uM	uM	uM	uM	uM	uM-1	uM-1	uM-1	uM-1	s-1	s-1	s-1	s-1	s-1	um2.s-1	um2.s-1	um2.s-1	um2.s-1	s	s	s
Fig.3B	18mm scale	1	2000	200	6.00E-06	1.00E-07	5.00E-09	3.50E-09	1.00E-06	1.00E-06	2.50E+02	2.50E+02	1.00E+04	5.00E+02	0.01	0.01	0	0	0.001	25000	80	1000	1000	0.1	1200	1200
	0.5mm scale	2	50	5	6.00E-06	1.00E-07	5.00E-09	3.50E-09	1.00E-06	1.00E-06	2.50E+02	2.50E+02	1.00E+04	5.00E+02	0.01	0.01	0	0	0	0.43	0	0.02	0.02	1	10000	10000
parameters for Fig4.ABC		3	50	5	6.00E-06	1.00E-07	5.00E-09	3.50E-09	1.00E-06	1.00E-06	2.50E+02	2.50E+02	1.00E+04	5.00E+02	0.01	0.01	0	0	0	0.3	0	0.3	0.3	1	600	600
		4	50	5	6.00E-06	1.00E-07	5.00E-09	3.50E-09	1.00E-06	1.00E-06	2.50E+02	2.50E+02	1.00E+04	5.00E+02	0.01	0.01	0	0	0	0.65	0	0.3	0.3	1	600	600
		5	50	5	6.00E-06	1.00E-07	5.00E-09	3.50E-09	1.00E-06	1.00E-06	2.50E+02	2.50E+02	1.00E+04	5.00E+02	0.01	0.01	0	0	0	1.39	0	0.3	0.3	1	600	600
		6	50	5	6.00E-06	1.00E-07	5.00E-09	3.50E-09	1.00E-06	1.00E-06	2.50E+02	2.50E+02	1.00E+04	5.00E+02	0.01	0.01	0	0	0	3	0	0.3	0.3	1	600	600
		7	50	5	6.00E-06	1.00E-07	5.00E-09	3.50E-09	1.00E-06	1.00E-06	2.50E+02	2.50E+02	1.00E+04	5.00E+02	0.01	0.01	0	0	0	6.46	0	0.3	0.3	0.96	600	600
		8	50	5	6.00E-06	1.00E-07	5.00E-09	3.50E-09	1.00E-06	1.00E-06	2.50E+02	2.50E+02	1.00E+04	5.00E+02	0.01	0.01	0	0	0	13.92	0	0.3	0.3	0.44	600	600
		9	50	5	6.00E-06	1.00E-07	5.00E-09	3.50E-09	1.00E-06	1.00E-06	2.50E+02	2.50E+02	1.00E+04	5.00E+02	0.01	0.01	0	0	0	30	0	0.3	0.3	0.2	600	600
		10	50	5	6.00E-06	1.00E-07	5.00E-09	3.50E-09	1.00E-06	1.00E-06	2.50E+02	2.50E+02	1.00E+04	5.00E+02	0.01	0.01	0	0	0	64.63	0	0.3	0.3	0.096	600	600
		11	50	5	6.00E-06	1.00E-07	5.00E-09	3.50E-09	1.00E-06	1.00E-06	2.50E+02	2.50E+02	1.00E+04	5.00E+02	0.01	0.01	0	0	0	139.25	0	0.3	0.3	0.044	600	600
		12	50	5	6.00E-06	1.00E-07	5.00E-09	3.50E-09	1.00E-06	1.00E-06	2.50E+02	2.50E+02	1.00E+04	5.00E+02	0.01	0.01	0	0	0	300	0	0.3	0.3	0.02	600	600
		13	50	5	6.00E-06	1.00E-07	5.00E-09	3.50E-09	1.00E-06	1.00E-06	2.50E+02	2.50E+02	1.00E+04	5.00E+02	0.01	0.01	0	0	0	0.3	0	0.65	0.65	1	600	600
		14	50	5	6.00E-06	1.00E-07	5.00E-09	3.50E-09	1.00E-06	1.00E-06	2.50E+02	2.50E+02	1.00E+04	5.00E+02	0.01	0.01	0	0	0	0.65	0	0.65	0.65	1	600	600
		15	50	5	6.00E-06	1.00E-07	5.00E-09	3.50E-09	1.00E-06	1.00E-06	2.50E+02	2.50E+02	1.00E+04	5.00E+02	0.01	0.01	0	0	0	1.39	0	0.65	0.65	1	600	600
		16	50	5	6.00E-06	1.00E-07	5.00E-09	3.50E-09	1.00E-06	1.00E-06	2.50E+02	2.50E+02	1.00E+04	5.00E+02	0.01	0.01	0	0	0	3	0	0.65	0.65	1	600	600
		17	50	5	6.00E-06	1.00E-07	5.00E-09	3.50E-09	1.00E-06	1.00E-06	2.50E+02	2.50E+02	1.00E+04	5.00E+02	0.01	0.01	0	0	0	6.46	0	0.65	0.65	0.96	600	600
		18	50	5	6.00E-06	1.00E-07	5.00E-09	3.50E-09	1.00E-06	1.00E-06	2.50E+02	2.50E+02	1.00E+04	5.00E+02	0.01	0.01	0	0	0	13.92	0	0.65	0.65	0.44	600	600
		19	50	5	6.00E-06	1.00E-07	5.00E-09	3.50E-09	1.00E-06	1.00E-06	2.50E+02	2.50E+02	1.00E+04	5.00E+02	0.01	0.01	0	0	0	30	0	0.65	0.65	0.2	600	600
		20	50	5	6.00E-06	1.00E-07	5.00E-09	3.50E-09	1.00E-06	1.00E-06	2.50E+02	2.50E+02	1.00E+04	5.00E+02	0.01	0.01	0	0	0	64.63	0	0.65	0.65	0.096	600	600
		21	50	5	6.00E-06	1.00E-07	5.00E-09	3.50E-09	1.00E-06	1.00E-06	2.50E+02	2.50E+02	1.00E+04	5.00E+02	0.01	0.01	0	0	0	139.25	0	0.65	0.65	0.044	600	600
		22	50	5	6.00E-06	1.00E-07	5.00E-09	3.50E-09	1.00E-06	1.00E-06	2.50E+02	2.50E+02	1.00E+04	5.00E+02	0.01	0.01	0	0	0	300	0	0.65	0.65	0.02	600	600
		23	50	5	6.00E-06	1.00E-07	5.00E-09	3.50E-09	1.00E-06	1.00E-06	2.50E+02	2.50E+02	1.00E+04	5.00E+02	0.01	0.01	0	0	0	0.3	0	1.39	1.39	1	600	600
		24	50	5	6.00E-06	1.00E-07	5.00E-09	3.50E-09	1.00E-06	1.00E-06	2.50E+02	2.50E+02	1.00E+04	5.00E+02	0.01	0.01	0	0	0	0.65	0	1.39	1.39	1	600	600
		25	50	5	6.00E-06	1.00E-07	5.00E-09	3.50E-09	1.00E-06	1.00E-06	2.50E+02	2.50E+02	1.00E+04	5.00E+02	0.01	0.01	0	0	0	1.39	0	1.39	1.39	1	600	600
		26	50	5	6.00E-06	1.00E-07	5.00E-09	3.50E-09	1.00E-06	1.00E-06	2.50E+02	2.50E+02	1.00E+04	5.00E+02	0.01	0.01	0	0	0	3	0	1.39	1.39	1	600	600
		27	50	5	6.00E-06	1.00E-07	5.00E-09	3.50E-09	1.00E-06	1.00E-06	2.50E+02	2.50E+02	1.00E+04	5.00E+02	0.01	0.01	0	0	0	6.46	0	1.39	1.39	0.96	600	600
		28	50	5	6.00E-06	1.00E-07	5.00E-09	3.50E-09	1.00E-06	1.00E-06	2.50E+02	2.50E+02	1.00E+04	5.00E+02	0.01	0.01	0	0	0	13.92	0	1.39	1.39	0.44	600	600
		29	50	5	6.00E-06	1.00E-07	5.00E-09	3.50E-09	1.00E-06	1.00E-06	2.50E+02	2.50E+02	1.00E+04	5.00E+02	0.01	0.01	0	0	0	30	0	1.39	1.39	0.2	600	600
		30	50	5	6.00E-06	1.00E-07	5.00E-09	3.50E-09	1.00E-06	1.00E-06	2.50E+02	2.50E+02	1.00E+04	5.00E+02	0.01	0.01	0	0	0	64.63	0	1.39	1.39	0.096	600	600
		31	50	5	6.00E-06	1.00E-07	5.00E-09	3.50E-09	1.00E-06	1.00E-06	2.50E+02	2.50E+02	1.00E+04	5.00E+02	0.01	0.01	0	0	0	139.25	0	1.39	1.39	0.044	600	600
		32	50	5	6.00E-06	1.00E-07	5.00E-09	3.50E-09	1.00E-06	1.00E-06	2.50E+02	2.50E+02	1.00E+04	5.00E+02	0.01	0.01	0	0	0	300	0	1.39	1.39	0.02	600	600
Fig4.D	no deg	33	50	5	6.00E-06	1.00E-07	5.00E-09	3.50E-09	1.00E-06	1.00E-06	2.50E+02	2.50E+02	1.00E+04	5.00E+02	0.01	0.01	0	0	0	0.43	0	0.02	0.02	1	9000	900
	with deg	34	50	5	6.00E-06	1.00E-07	5.00E-09	3.50E-09	1.00E-06	1.00E-06	2.50E+02	2.50E+02	1.00E+04	5.00E+02	0.01	0.01	0	0.0009	0	0.43	0	0.02	0.02	1	9000	900

Note: these parameter values were used for generating results for the repressed network in Fig. 3 and 4 of the main text. To run these values, first download the accompanying ‘*param.txt*’ file in *Supplementary Information*. Next, double-click the ‘*simul.pl*’ file containing the script to execute (or call it from a command prompt), and follow the prompts (‘p’ for uploading the values from the file, and twice <ENTER> to get default outputs and output location).

9. *Output options: contents and location*

After defining the input data, the user is asked to specify which species require outputs by typing their names, separated by commas. As a reminder to the user, a complete list of variables is provided. The default setting for this is selected by pressing <ENTER> and generates outputs for repressor proteins A, B and C (equivalent to typing 'Ap,Bp,Cp').

The amount of data to be generated for each output species is decided by choosing the period of output. By default, only the data for the last timestep is output, giving a snapshot of the system at the end of the simulation, but the user can specify any other period.

For each species and each timestep where output should be generated, two datasets are produced: one carries the concentration values of the given species at each position in the system, the other carries a normalized value of the previous dataset (the sum of the values over all minislabs is equal to 1). This second dataset is useful in comparing profiles for sets where absolute values differ by more than an order of magnitude.

Each dataset is stored in a separate text file carrying the species' name after the type of data the file contains (normalized 'norm' or absolute '').
Within each of these text files, each line of data consists of a tab-delimited series of concentration values of the given species corresponding to increasing positions in the system (from first to last minislab). The corresponding timelapse between two lines is equal to the period of data retrieval specified by the user.

In addition, and independently of the mode used to input parameter values (manual, default or read from a file), all parameter sets used to run the simulations are copied into a file called 'param.txt' in the same location as the previous files. This file can then be edited and used to re-run with modified parameter values.

All output results (species concentrations and parameter file) are placed in a single common folder for each time the script is run. The user is prompted to give a name to the folder, which is then placed in the same location as the perl script.

Note: For OS X, the output folders created may have insufficient privileges. Make sure folders have 'read and write' privileges from "Show Info" (cmd-I).

However, if the user chooses to use a default location (by typing <ENTER>), a series of folders are created under a single 'sim-results' folder, and the output folder for this run is placed inside according to the number of parameter sets specified (single-run or multiple-runs), and the period of output (single-time or timecourse). The naming scheme is as follows: an X followed by a random number (between 0 and 10000) (see example below).

Example:

For a single run, following species Ap and Bp with outputs for every 5 minutes of simulation, 5 textfiles were generated :

single-run/timecourse/X1234/Ap.txt
single-run/timecourse/X1234/normAp.txt
single-run/timecourse/X1234/Bp.txt
single-run/timecourse/X1234/normBp.txt
single-run/timecourse/X1234/param.txt

The ‘norm’ prefix, indicates that the data file carries the normalised values.

For these output files (except param.txt), each consecutive row of output data will correspond to a complete set of concentration values, for all minislab positions, for a particular timepoint, 5 minutes after the previous timepoint (previous row).

****

Appendix A: Quantitated Western blot data

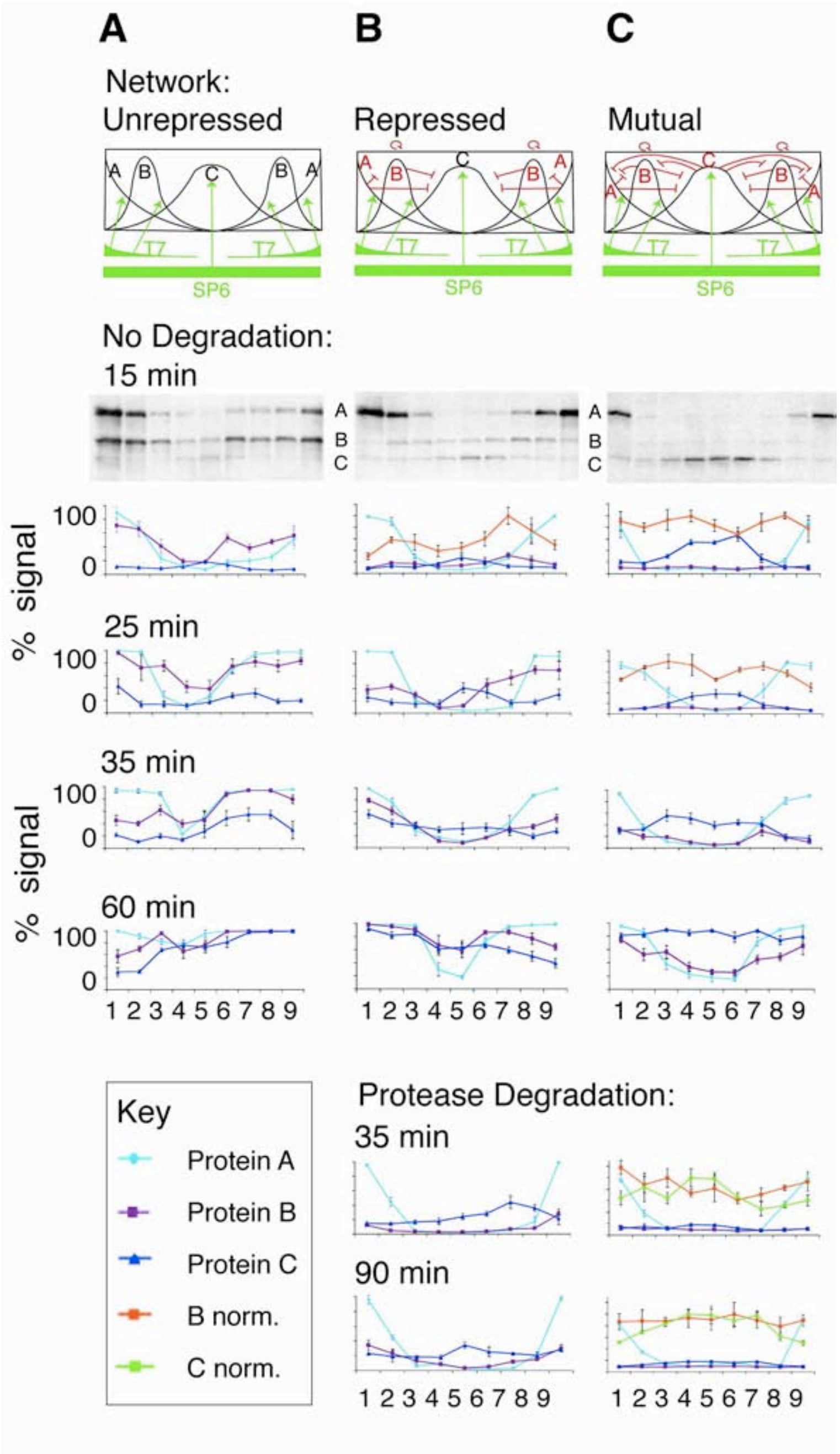

Supplementary Fig S3 Quantified data for the Western blots shown in Fig 2 of the main text. Blots were scanned and quantitated using Molecular Dynamics ImageQuant v1.2. Above: ('No Degradation') data are shown for the three output species, proteins A, B and C, taken at time intervals between 15 and 60 minutes, for the three different gene networks tested ('Unrepressed', 'Repressed and 'Mutual'). For each band in the blots, intensity measurements were taken at three points, and mean values were plotted (+/- 1 s.d.). For clarity, selected B,C signals are also shown with their values normalised to the maximum signal of the same protein within the same blot (orange/green series). For the 15 minute timepoint, sample raw western blots are also shown. Below: ('Protease Degradation') Incubations were carried out in the presence of Factor Xa protease degradation and data are shown for the three output species, proteins A, B and C, taken at time intervals of 35 or 90 minutes, for two different gene networks tested ('Repressed and 'Mutual').

Appendix B

Step-by-step construction of a transcription-translation chamber reaction

The section that follows describes how to construct a transcription-translation chamber for gene network reactions from PCR-coated magnetic beads. Reactions are carried out on sterile petridishes (a fresh one for each new experiment). The Petri dish has a reaction chamber built on its base, using adhesive hybriwell strips. This chamber is built over a plasticine-filled dish, containing magnets. It should be noted, that we have also successfully built chambers by drilling holes for magnets in an acrylic base; this alternative can provide a more robust and regular support. Chamber construction is guided by a printed template, placed over the magnets. The chamber is filled with a transcription-translation reaction, containing ultra-low melting point agarose gel. DNA-coated beads are dispensed into this mixture, over discrete magnetic positions. Finally, T7 polymerase is pipetted at either end of the chamber to begin localised transcription.

Overall chamber design

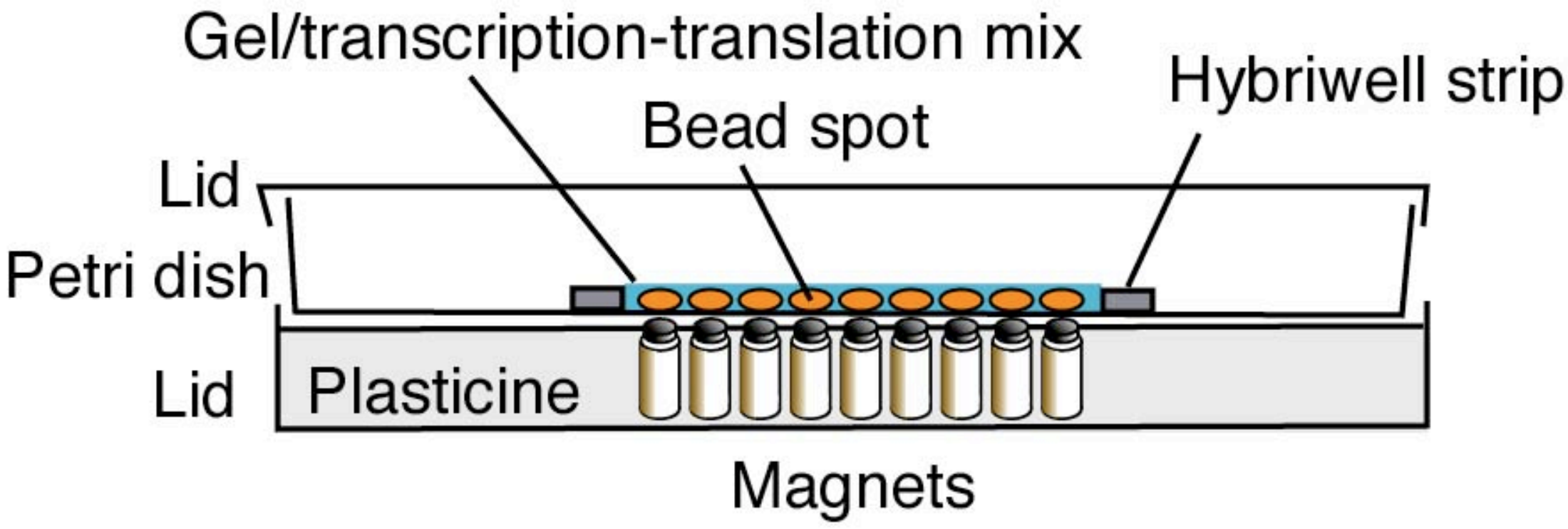

Materials:

Sterile Petri dishes treated for cell culture (Nunclon, Nalge-Nunc, Cat. #150350)

Stirrer bar magnets, 8 × 1.5 mm (VWR International, Germany, Cat. #4429025)

Adhesive Hybriwell chambers (Sigma, Cat. #H1159-100EA)

Clean scissors

Adhesive tape

RNaseZAP (Sigma, Cat. #R-2020).

Dynabeads Kilobase Binder Kit (Dynal; Cat. #601.01).

TNT wheat germ kit (Promega, Cat. #L4130, #L4140)

Ultra low melting point agarose solution (Sigma, Cat. #A2576)

Anti-M2 FLAG antibody (Sigma, Cat. #F3165).

1. Place a 7 mm deep layer of light-coloured modelling clay (e.g. white plasticine) in the lid of a plastic 90 mm diameter Petri dish. It is not necessary to fill the entire lid but the layer must be sufficiently broad to support the base of a second petridish (see picture below). A 3-prong shape is effective and can be flattened smooth, using the base of another dish.

1.

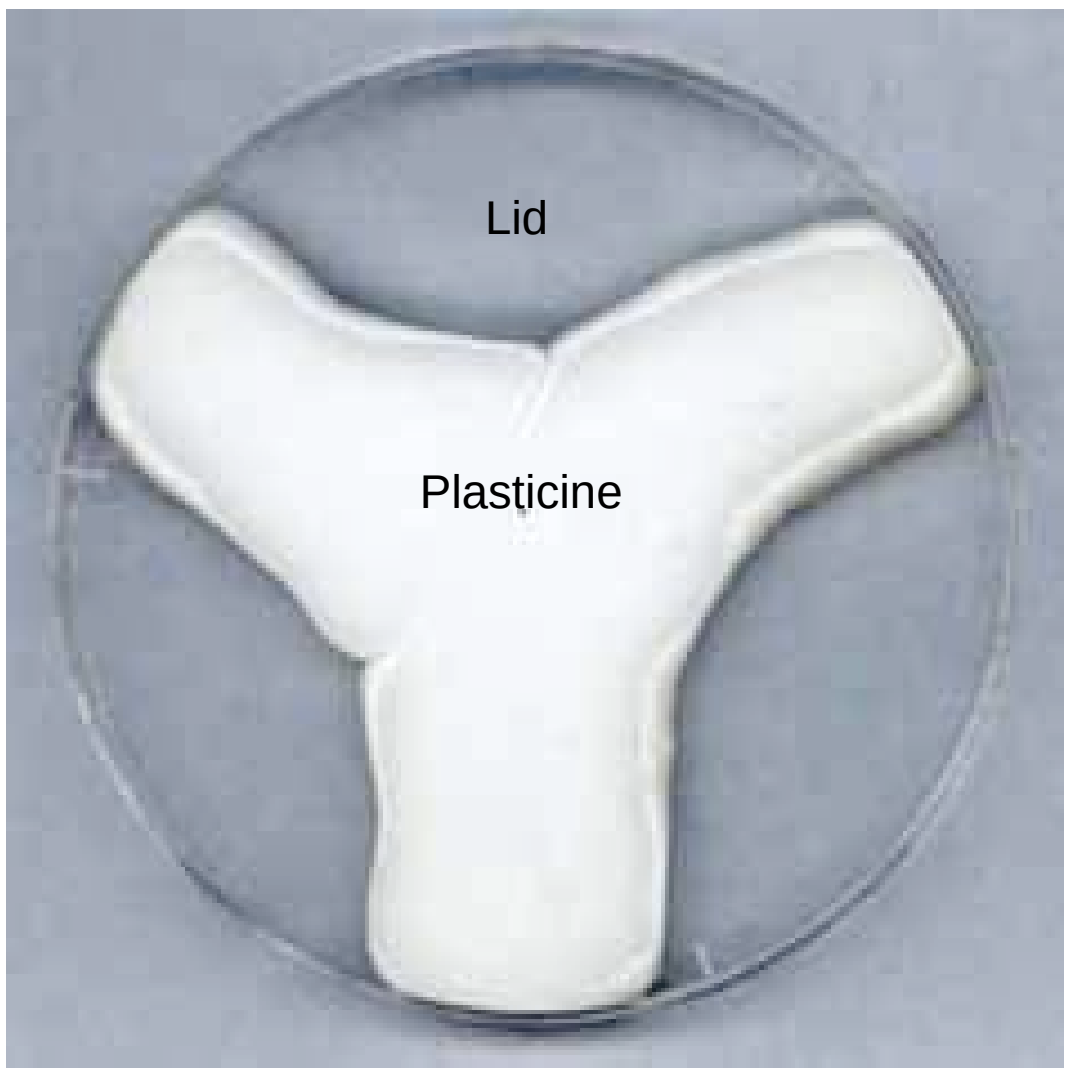

2.

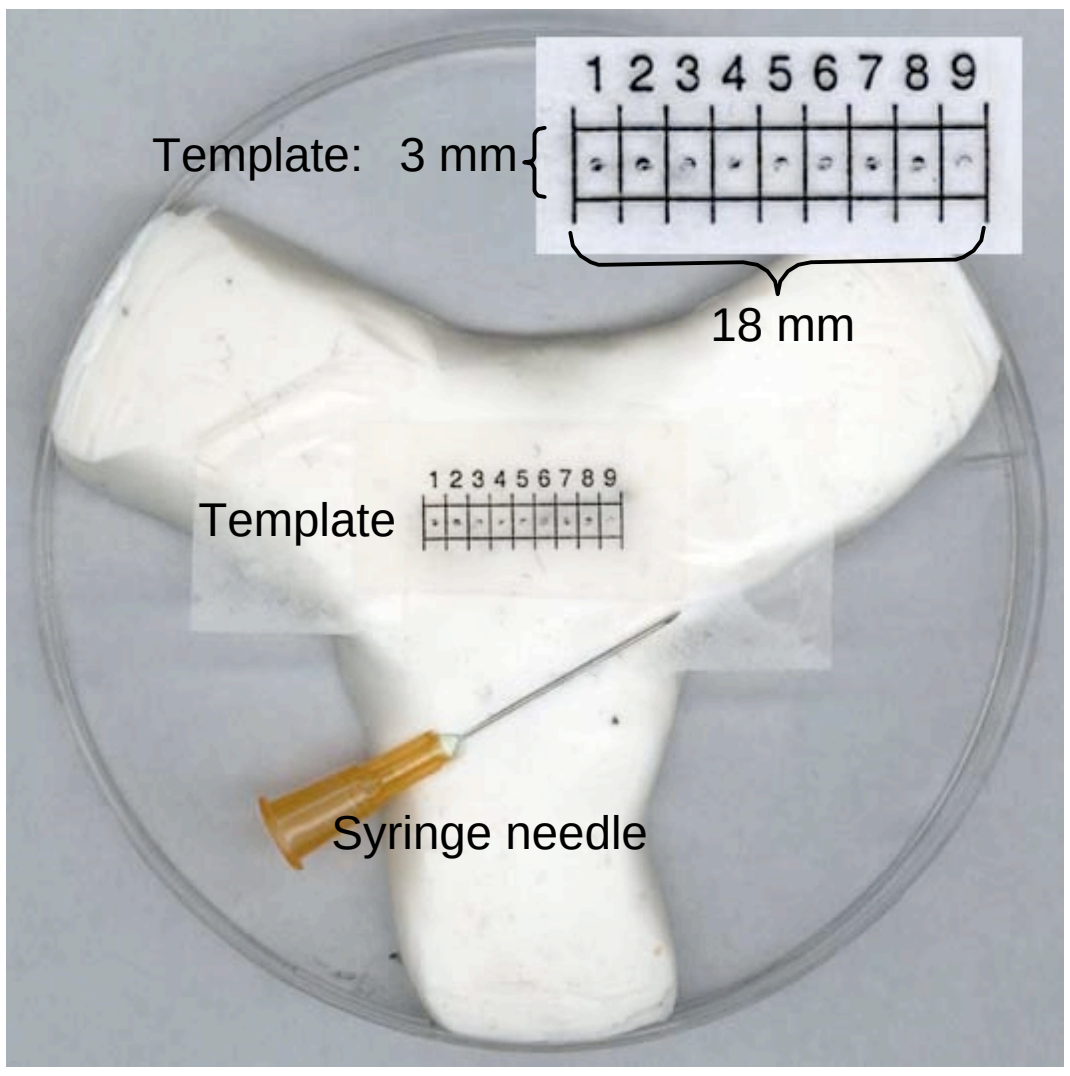

3.

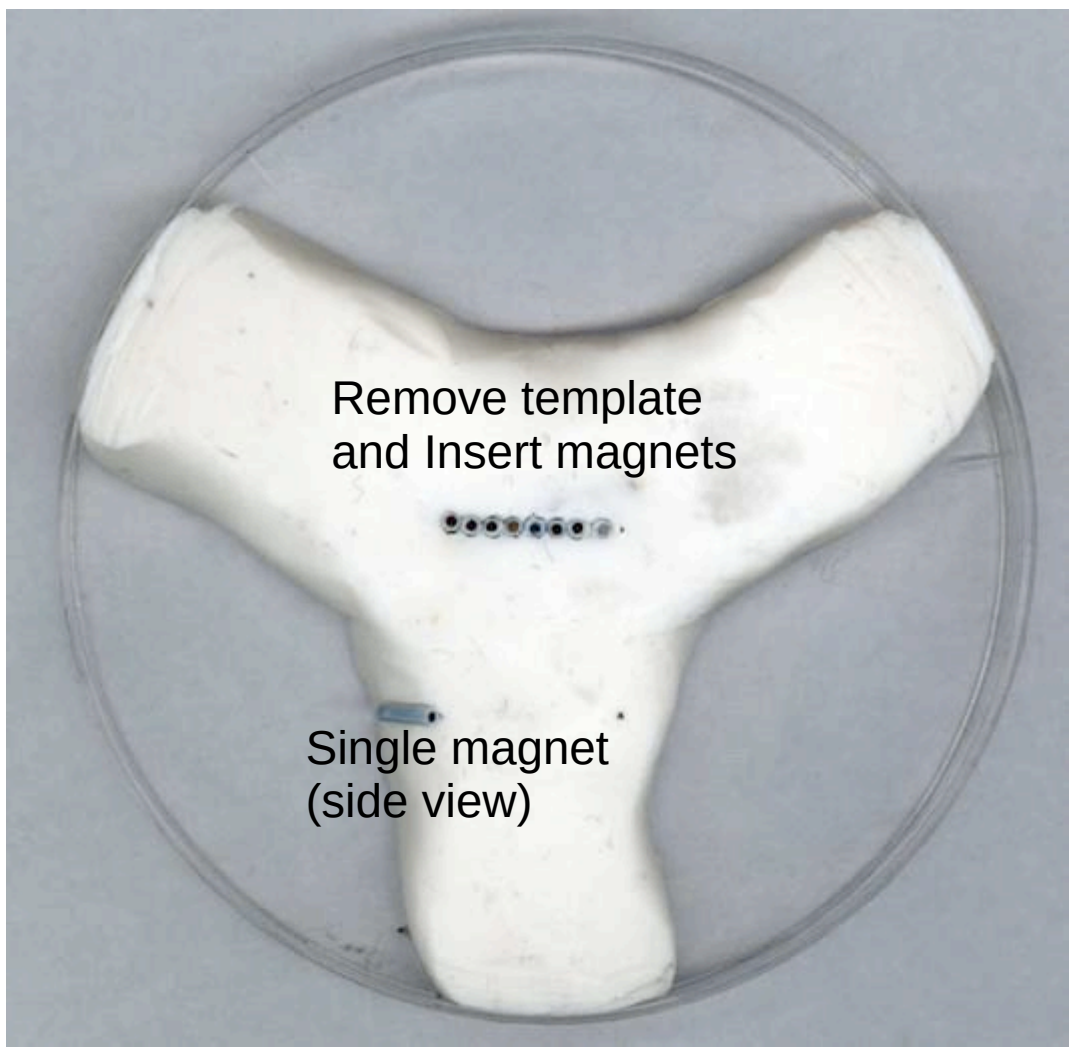

4.

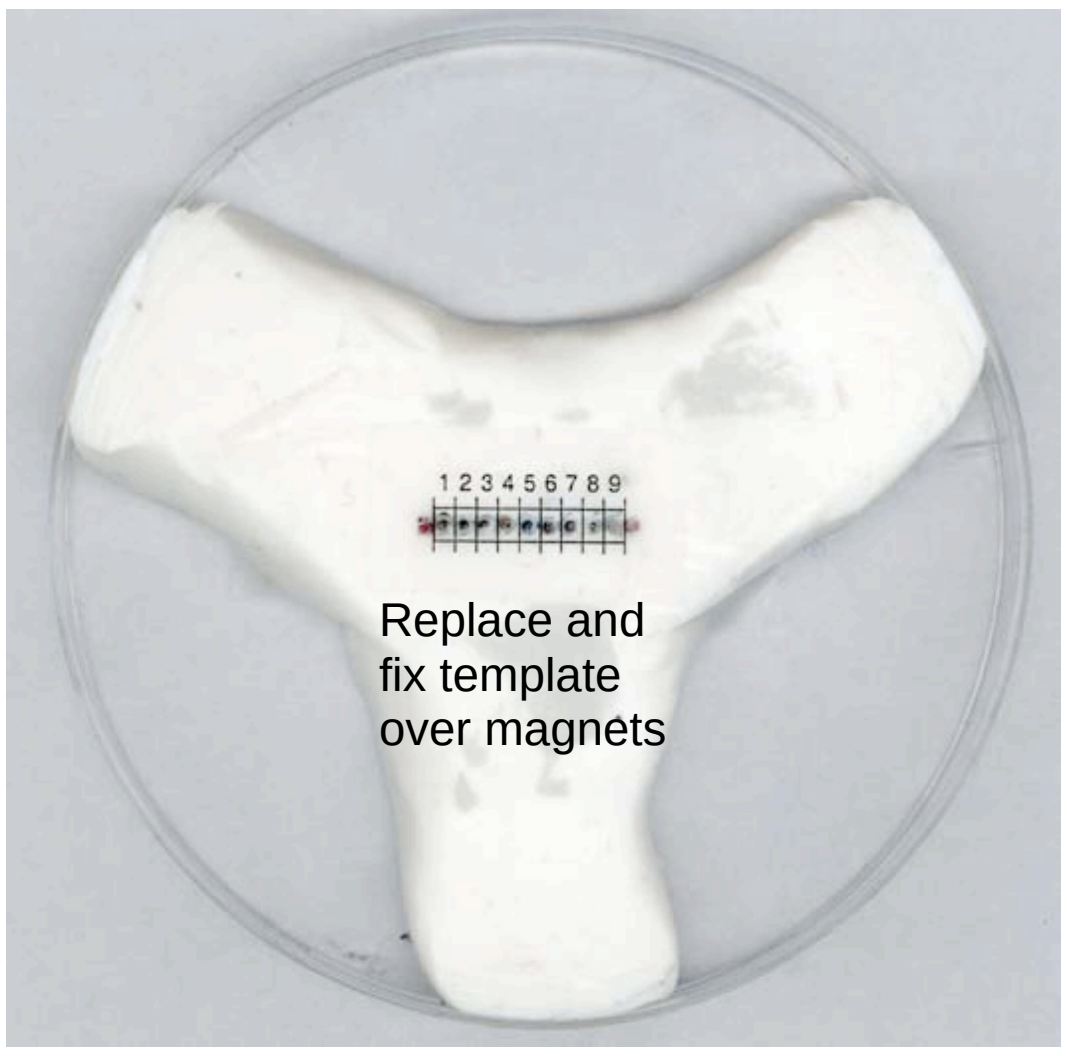

2. Print a template for the magnetic chamber on an acetate sheet and place this on the plasticine. Use a fine needle to pierce the template and make holes in the plasticine in which to insert the magnets.

3. Insert magnets into the plasticine, as shown, using the needle holes from *Step 2.* as a guide. It is convenient to use 8 x 1.5 mm stirrer-bar magnets. Magnets should be clipped with scissors to expose the metal below the plastic coat, allowing the magnet to make a flush surface with the plasticine bed. It is important that identical magnet poles face the same side (e.g. all North), otherwise magnetic beads do not form discrete spots and get distributed along field lines. To avoid errors, the magnetic field may be visualised by putting a piece of paper over the array and spreading iron filings over the area. An extra

pair of magnets should be added at each end of the array to avoid edge effects (edge spots are otherwise more diffuse as the magnetic field spreads outwards, away from the line of magnets).

4. Replace the acetate template over the magnet array and fix with adhesive tape.

5. Take a sterile Petri dish with lid, place it over the plasticine-magnet base and fix in place with adhesive tape. Note the final layout of dishes and chamber in the schematic figure at the beginning of this section ("Overall chamber design").

6. Construct the edges of the chamber using strips cut from adhesive Hybriwell chambers (see picture below). Two layers of strips are sufficient to make a chamber 1 mm deep. Note: all materials and surroundings (e.g. gloves, scissors, bench) should be RNase-free and is it useful to wipe clean with RNaseZAP.

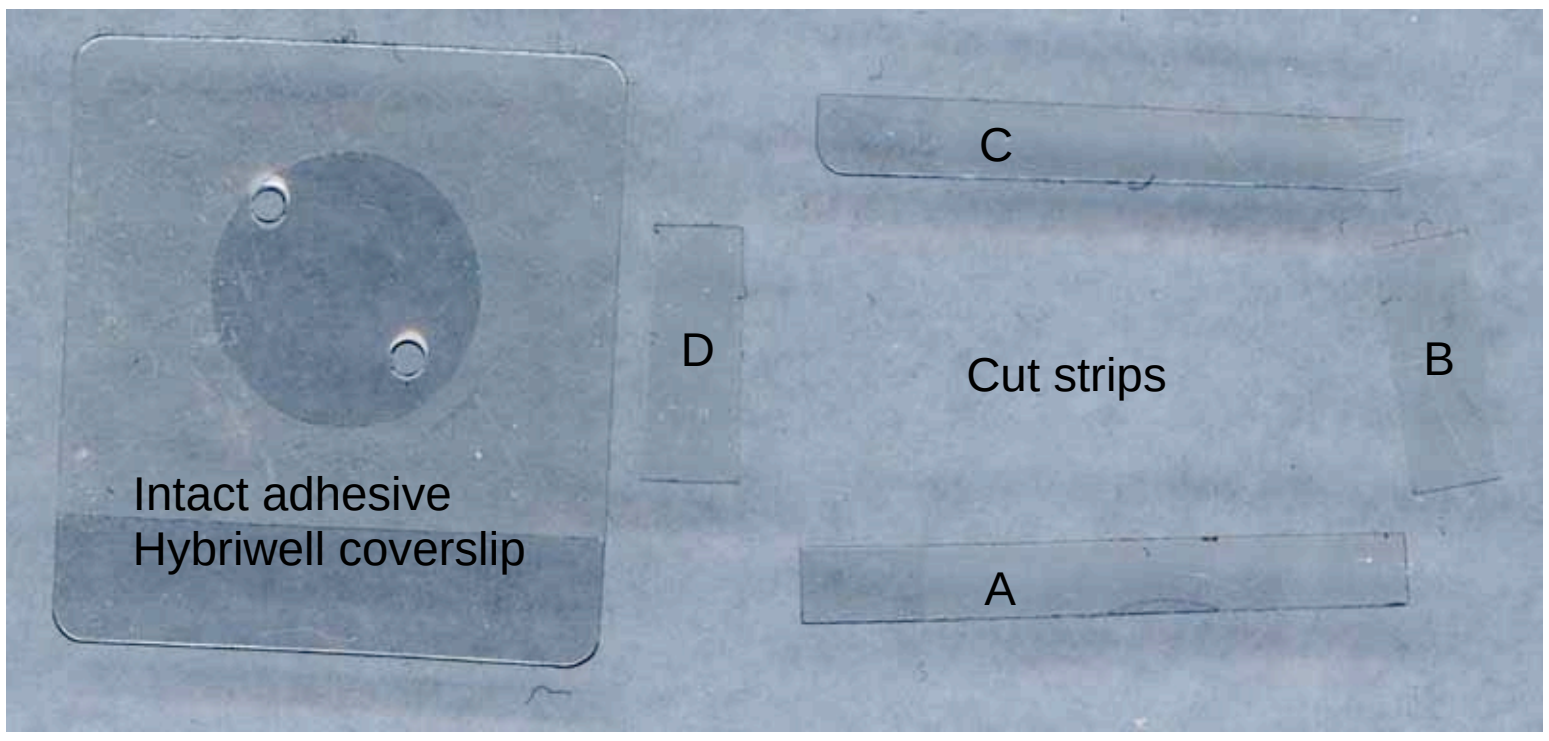

Cut the Hybriwell coverslip into approximately 3 mm wide strips with scissors.

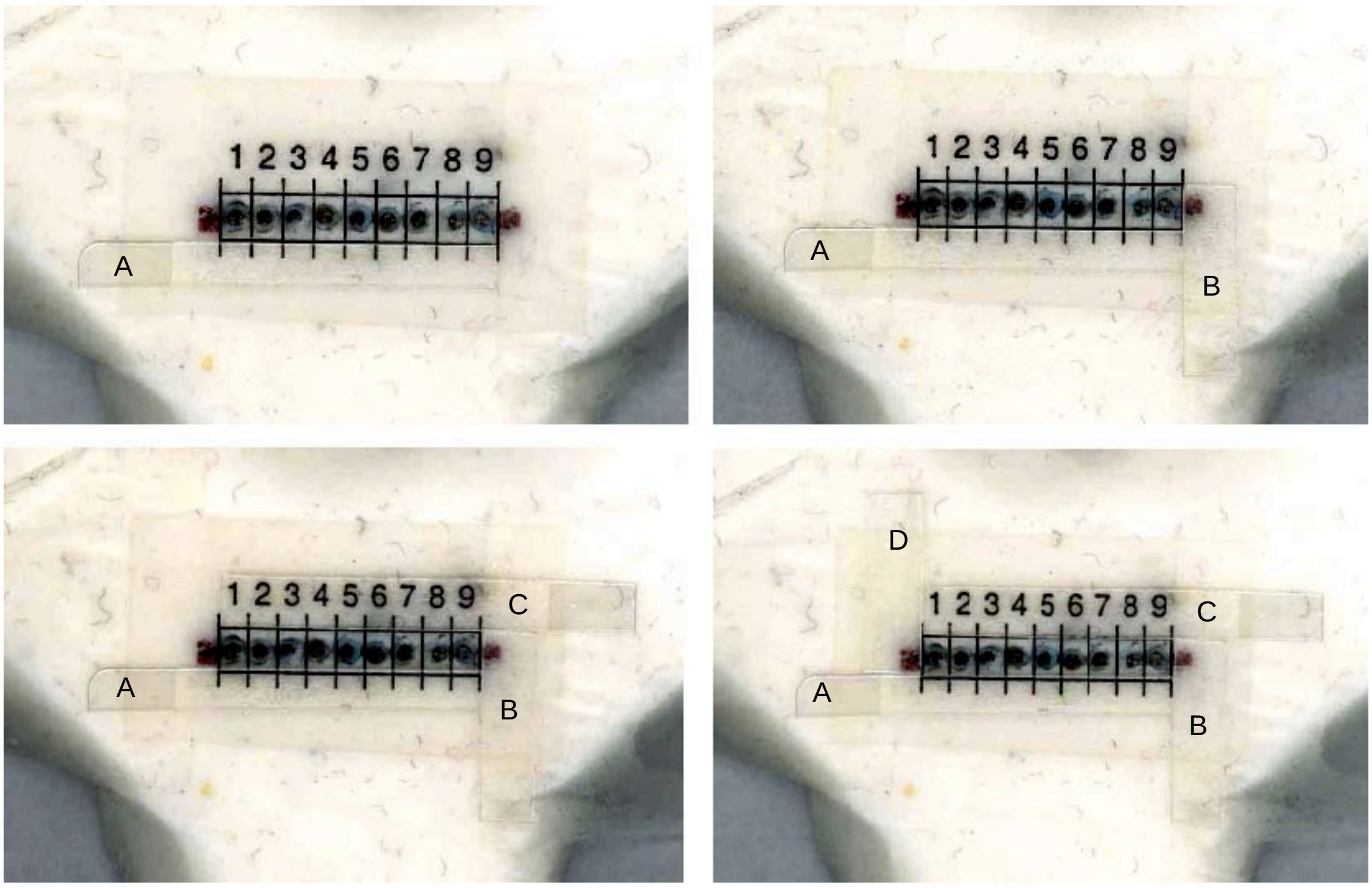

Add Hybriwell strips to form a chamber: A then B then C then D.

The finished chamber on the base of the sterile Petri dish should look like this (although two layers of hybriwell strips are preferred):

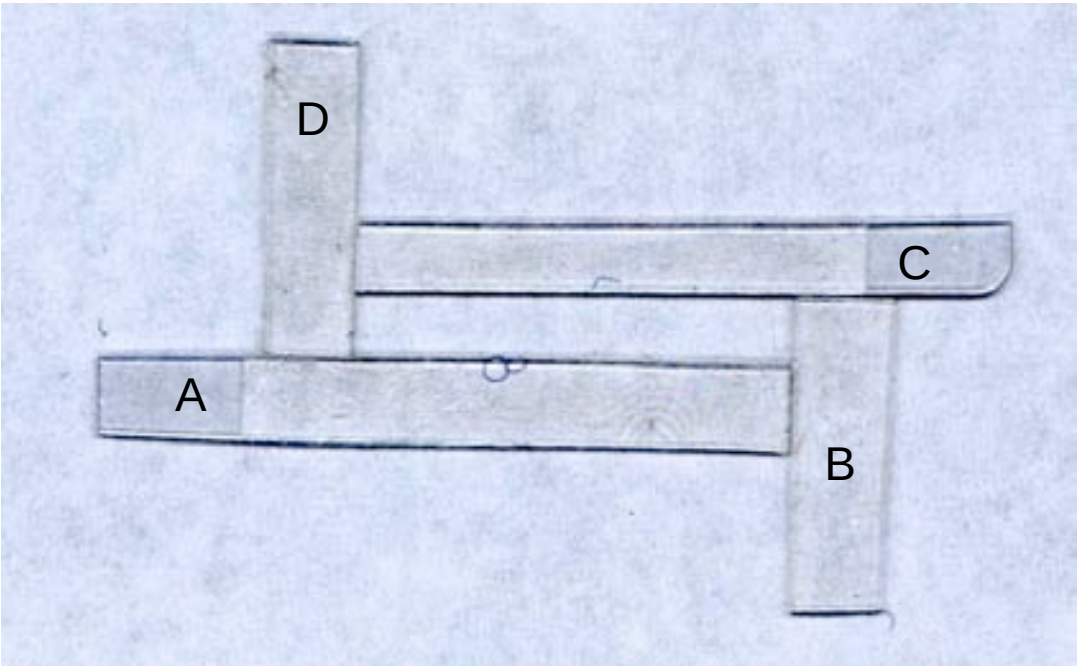

7. Coat paramagnetic beads with PCR DNA (one primer biotinylated) using a Dynabeads Kilobase Binder Kit. Typically, gene A was used at 800 fmol per 10 μl beads, resuspended in 8 μl water; 200 fmol gene B and 140 fmol gene C were combined with 20 μl beads, and resuspended in 20 μl water.

8. Prepare a transcription-translation mixture using a TNT wheat germ kit:

2.5 μl water
20 μl TNT wheatgerm extract
1.2 μl TNT Reaction Buffer
0.6 μl amino acid mixture (1 mM)
1.2 μl RNasin (Promega; optional)
0.5 μl SP6 polymerase.
28 μl ultra low melting point agarose solution (melt 1.5 % (w/v) in boiling water, equilibrate in a 30°C water bath for at least 10 min, mixing well before use).

Add the agarose last, mix and go to *Step 9*. immediately.

9. Dispense 54 μl of transcription-translation mixture per 18 x 3 x 1 mm chamber. Immediately add resuspended (vortexed) magnetic beads by pipetting 1 or 0.5 μl volumes gently over the base of the chamber, as close to each magnetic spot as possible. The pipetting action is similar to loading a gel.

10. After the beads are arrayed, simultaneously inject 0.5 μl of T7 polymerase at either chamber end, using an appropriately loaded 0-10 μl multipipette. This is the start point of the experiment (see picture that follows).

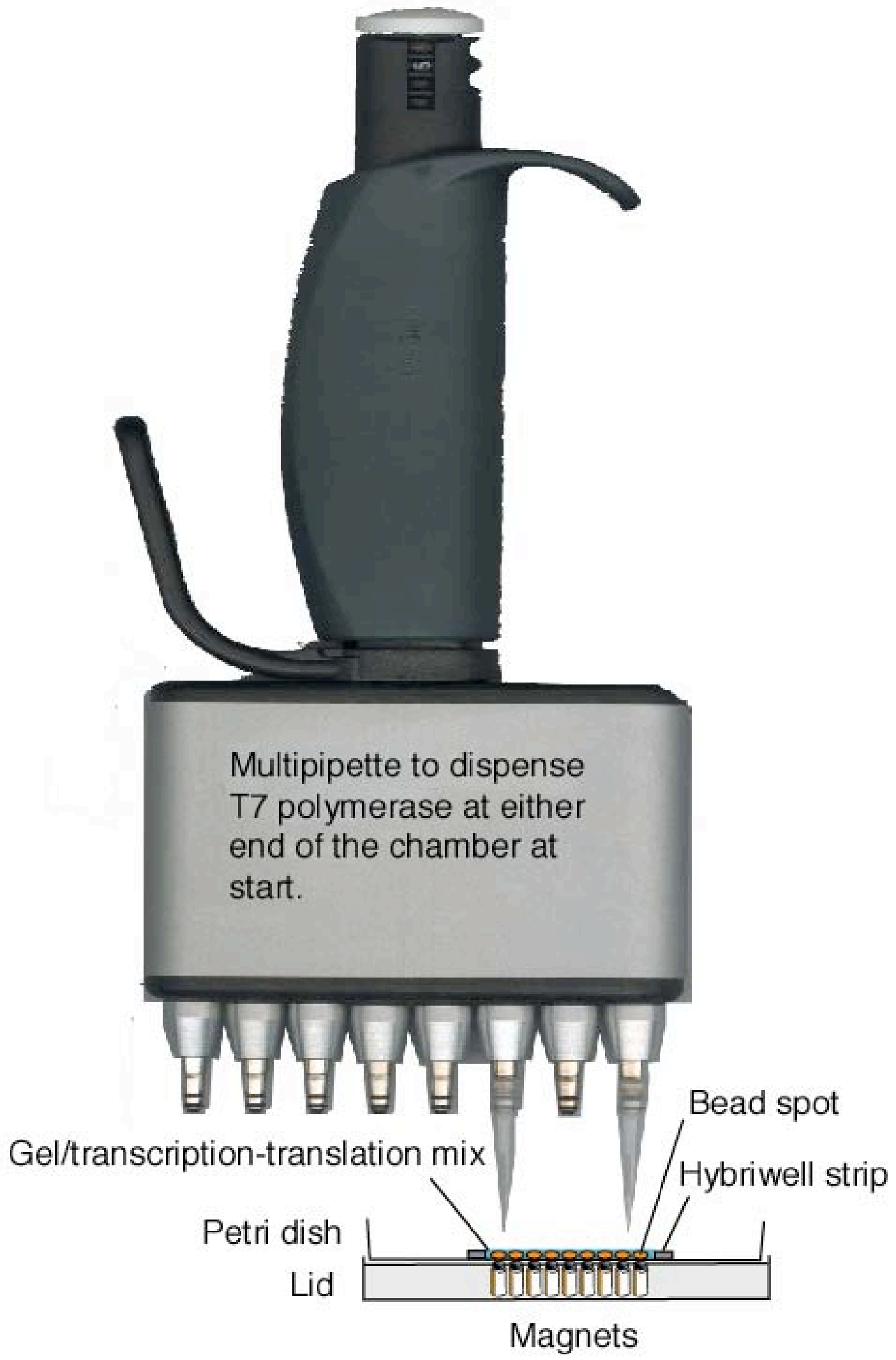

The chamber may be incubated at room temperature (25˚C). To minimise evaporation, tissues moistened with distilled water may be placed inside the Petri dish, avoiding contact with the transcription-translation chamber itself.

11. At the chosen end point (e.g. 15 to 60 min), transfer the entire chamber to 4˚C, to gel for 35 min. The ultra-low melting point agarose will gel at temperatures below 17˚C.

12. Gel slices may be cut with a razorblade (make a single cut, following a line on the acetate template as a guide). Aspirate the gel with a P10 Gilson pipette, set to a nominal 10 μl volume. Mix the gel samples with 10 μl SDS-loading buffer and analyse by SDS-PAGE, Western-blotting and ECL, with anti-M2 FLAG antibody.

Note:The wheat germ extract is autofluorescent and prevents direct fluorescence readout (eg GFP). However, we anticipate that with *E. coli* extract and using fluorescein digalactoside/lacZ as a reporter it should be possible to use a fluorescent scanner to obtain output signals *in situ.*
