# Supplementary material for: Engineering Gene Networks to Emulate Drosophila Embryonic Pattern Formation
Source: PLoS Biol. 2005 Feb 22;3(3):e64. doi: 10.1371/journal.pbio.0030064 (PMC1044831; doi:10.1371/journal.pbio.0030064)
Supplement: Supplementary file 2 [file pbio.0030064.sd002.doc]

\# run	slablength	minislablength	slabvol	dnaA	dnaB	dnaC	X0	Y0	KassX	KassY	KassAp	KassBp	ktr	ktl	kdegr	kdegp	kX	DX0	Dres	Dm	Dp	timestep	run-time	sampleperiod

\# parameters for Fig.3B, repressed network, 18mm scale

1	2000	200	6.00E-06	1.00E-07	5.00E-09	3.50E-09	1.00E-06	1.00E-06	2.50E+02	2.50E+02	1.00E+04	5.00E+02	0.01	0.01	0	0	0.001	25000	80	1000	1000	0.1	1200	1200

\# parameters for Fig.3B, repressed network, 0.5mm scale

2	50	5	6.00E-06	1.00E-07	5.00E-09	3.50E-09	1.00E-06	1.00E-06	2.50E+02	2.50E+02	1.00E+04	5.00E+02	0.01	0.01	0	0	0	0.43	0	0.02	0.02	1	10000	10000

\# parameters for Fig4.ABC

3	50	5	6.00E-06	1.00E-07	5.00E-09	3.50E-09	1.00E-06	1.00E-06	2.50E+02	2.50E+02	1.00E+04	5.00E+02	0.01	0.01	0	0	0	0.3	0	0.3	0.3	1	600	600

4	50	5	6.00E-06	1.00E-07	5.00E-09	3.50E-09	1.00E-06	1.00E-06	2.50E+02	2.50E+02	1.00E+04	5.00E+02	0.01	0.01	0	0	0	0.646330407	0	0.3	0.3	1	600	600

5	50	5	6.00E-06	1.00E-07	5.00E-09	3.50E-09	1.00E-06	1.00E-06	2.50E+02	2.50E+02	1.00E+04	5.00E+02	0.01	0.01	0	0	0	1.39247665	0	0.3	0.3	1	600	600

6	50	5	6.00E-06	1.00E-07	5.00E-09	3.50E-09	1.00E-06	1.00E-06	2.50E+02	2.50E+02	1.00E+04	5.00E+02	0.01	0.01	0	0	0	3	0	0.3	0.3	1	600	600

7	50	5	6.00E-06	1.00E-07	5.00E-09	3.50E-09	1.00E-06	1.00E-06	2.50E+02	2.50E+02	1.00E+04	5.00E+02	0.01	0.01	0	0	0	6.46330407	0	0.3	0.3	0.96	600	600

8	50	5	6.00E-06	1.00E-07	5.00E-09	3.50E-09	1.00E-06	1.00E-06	2.50E+02	2.50E+02	1.00E+04	5.00E+02	0.01	0.01	0	0	0	13.9247665	0	0.3	0.3	0.44	600	600

9	50	5	6.00E-06	1.00E-07	5.00E-09	3.50E-09	1.00E-06	1.00E-06	2.50E+02	2.50E+02	1.00E+04	5.00E+02	0.01	0.01	0	0	0	30	0	0.3	0.3	0.2	600	600

10	50	5	6.00E-06	1.00E-07	5.00E-09	3.50E-09	1.00E-06	1.00E-06	2.50E+02	2.50E+02	1.00E+04	5.00E+02	0.01	0.01	0	0	0	64.6330407	0	0.3	0.3	0.096	600	600

11	50	5	6.00E-06	1.00E-07	5.00E-09	3.50E-09	1.00E-06	1.00E-06	2.50E+02	2.50E+02	1.00E+04	5.00E+02	0.01	0.01	0	0	0	139.247665	0	0.3	0.3	0.044	600	600

12	50	5	6.00E-06	1.00E-07	5.00E-09	3.50E-09	1.00E-06	1.00E-06	2.50E+02	2.50E+02	1.00E+04	5.00E+02	0.01	0.01	0	0	0	300	0	0.3	0.3	0.02	600	600

13	50	5	6.00E-06	1.00E-07	5.00E-09	3.50E-09	1.00E-06	1.00E-06	2.50E+02	2.50E+02	1.00E+04	5.00E+02	0.01	0.01	0	0	0	0.3	0	0.646330407	0.646330407	1	600	600

14	50	5	6.00E-06	1.00E-07	5.00E-09	3.50E-09	1.00E-06	1.00E-06	2.50E+02	2.50E+02	1.00E+04	5.00E+02	0.01	0.01	0	0	0	0.646330407	0	0.646330407	0.646330407	1	600	600

15	50	5	6.00E-06	1.00E-07	5.00E-09	3.50E-09	1.00E-06	1.00E-06	2.50E+02	2.50E+02	1.00E+04	5.00E+02	0.01	0.01	0	0	0	1.39247665	0	0.646330407	0.646330407	1	600	600

16	50	5	6.00E-06	1.00E-07	5.00E-09	3.50E-09	1.00E-06	1.00E-06	2.50E+02	2.50E+02	1.00E+04	5.00E+02	0.01	0.01	0	0	0	3	0	0.646330407	0.646330407	1	600	600

17	50	5	6.00E-06	1.00E-07	5.00E-09	3.50E-09	1.00E-06	1.00E-06	2.50E+02	2.50E+02	1.00E+04	5.00E+02	0.01	0.01	0	0	0	6.46330407	0	0.646330407	0.646330407	0.96	600	600

18	50	5	6.00E-06	1.00E-07	5.00E-09	3.50E-09	1.00E-06	1.00E-06	2.50E+02	2.50E+02	1.00E+04	5.00E+02	0.01	0.01	0	0	0	13.9247665	0	0.646330407	0.646330407	0.44	600	600

19	50	5	6.00E-06	1.00E-07	5.00E-09	3.50E-09	1.00E-06	1.00E-06	2.50E+02	2.50E+02	1.00E+04	5.00E+02	0.01	0.01	0	0	0	30	0	0.646330407	0.646330407	0.2	600	600

20	50	5	6.00E-06	1.00E-07	5.00E-09	3.50E-09	1.00E-06	1.00E-06	2.50E+02	2.50E+02	1.00E+04	5.00E+02	0.01	0.01	0	0	0	64.6330407	0	0.646330407	0.646330407	0.096	600	600

21	50	5	6.00E-06	1.00E-07	5.00E-09	3.50E-09	1.00E-06	1.00E-06	2.50E+02	2.50E+02	1.00E+04	5.00E+02	0.01	0.01	0	0	0	139.247665	0	0.646330407	0.646330407	0.044	600	600

22	50	5	6.00E-06	1.00E-07	5.00E-09	3.50E-09	1.00E-06	1.00E-06	2.50E+02	2.50E+02	1.00E+04	5.00E+02	0.01	0.01	0	0	0	300	0	0.646330407	0.646330407	0.02	600	600

23	50	5	6.00E-06	1.00E-07	5.00E-09	3.50E-09	1.00E-06	1.00E-06	2.50E+02	2.50E+02	1.00E+04	5.00E+02	0.01	0.01	0	0	0	0.3	0	1.39247665	1.39247665	1	600	600

24	50	5	6.00E-06	1.00E-07	5.00E-09	3.50E-09	1.00E-06	1.00E-06	2.50E+02	2.50E+02	1.00E+04	5.00E+02	0.01	0.01	0	0	0	0.646330407	0	1.39247665	1.39247665	1	600	600

25	50	5	6.00E-06	1.00E-07	5.00E-09	3.50E-09	1.00E-06	1.00E-06	2.50E+02	2.50E+02	1.00E+04	5.00E+02	0.01	0.01	0	0	0	1.39247665	0	1.39247665	1.39247665	1	600	600

26	50	5	6.00E-06	1.00E-07	5.00E-09	3.50E-09	1.00E-06	1.00E-06	2.50E+02	2.50E+02	1.00E+04	5.00E+02	0.01	0.01	0	0	0	3	0	1.39247665	1.39247665	1	600	600

27	50	5	6.00E-06	1.00E-07	5.00E-09	3.50E-09	1.00E-06	1.00E-06	2.50E+02	2.50E+02	1.00E+04	5.00E+02	0.01	0.01	0	0	0	6.46330407	0	1.39247665	1.39247665	0.96	600	600

28	50	5	6.00E-06	1.00E-07	5.00E-09	3.50E-09	1.00E-06	1.00E-06	2.50E+02	2.50E+02	1.00E+04	5.00E+02	0.01	0.01	0	0	0	13.9247665	0	1.39247665	1.39247665	0.44	600	600

29	50	5	6.00E-06	1.00E-07	5.00E-09	3.50E-09	1.00E-06	1.00E-06	2.50E+02	2.50E+02	1.00E+04	5.00E+02	0.01	0.01	0	0	0	30	0	1.39247665	1.39247665	0.2	600	600

30	50	5	6.00E-06	1.00E-07	5.00E-09	3.50E-09	1.00E-06	1.00E-06	2.50E+02	2.50E+02	1.00E+04	5.00E+02	0.01	0.01	0	0	0	64.6330407	0	1.39247665	1.39247665	0.096	600	600

31	50	5	6.00E-06	1.00E-07	5.00E-09	3.50E-09	1.00E-06	1.00E-06	2.50E+02	2.50E+02	1.00E+04	5.00E+02	0.01	0.01	0	0	0	139.247665	0	1.39247665	1.39247665	0.044	600	600

32	50	5	6.00E-06	1.00E-07	5.00E-09	3.50E-09	1.00E-06	1.00E-06	2.50E+02	2.50E+02	1.00E+04	5.00E+02	0.01	0.01	0	0	0	300	0	1.39247665	1.39247665	0.02	600	600

\## parameters for Fig4.D

\# without degradation

33	50	5	6.00E-06	1.00E-07	5.00E-09	3.50E-09	1.00E-06	1.00E-06	2.50E+02	2.50E+02	1.00E+04	5.00E+02	0.01	0.01	0	0	0	0.43	0	0.02	0.02	1	9000	900

\# with degradation

34	50	5	6.00E-06	1.00E-07	5.00E-09	3.50E-09	1.00E-06	1.00E-06	2.50E+02	2.50E+02	1.00E+04	5.00E+02	0.01	0.01	0	0.0009	0	0.43	0	0.02	0.02	1	9000	900
