# Supplementary material for: Engineering Gene Networks to Emulate Drosophila Embryonic Pattern Formation
Source: PLoS Biol. 2005 Feb 22;3(3):e64. doi: 10.1371/journal.pbio.0030064 (PMC1044831; doi:10.1371/journal.pbio.0030064)
Supplement: Supplementary file 3 [file pbio.0030064.sd003.doc]

\# June 18th,2004 rewrite of October 8th, 2003

#############################

\# description and purpose

###########################################################################################################################

\# this script allows for the simulation of a deterministic and discrete spatial model of the following system :

\# 2 transcription activators acting on 3 target genes each of which codes for repressors regulating these genes in a variable manner.

\# In the case of the repressed system, the interactions are the following :

\# gene A is unregulated, genes B and C are both repressed by repressors A and B

\# the aim of this model is to analyse the formation of the following spatial and temporal pattern :

\# 2 peaks of B protein on either side of a single peak of C protein, while A is highly expressed only at the ends

\# interest lies in the broadness of the peaks and their lifetime for given sets of parameter values......

\# as is the case in the experimental set-up, system space is divided into 9 slabs (here, subdivided further into mini-slabs)

###########################################################################################################################

\# prompt user for network type

$nw="false";

while ($nw eq "false") {

print "\n\nwhich network should be simulated : \n\trepressed ?...type r\n\tunrepressed ?...type u\n\tmutually repressed ?...type m\n\tcustom ?...type c\n\n";

chomp ($network=<STDIN>);

if ($network eq "r" || $network eq "u" || $network eq "m" || $network eq "c") {$nw="true"}

}

\# full list of parameters with default values, system variables and output options

@species=("X","Y","Am","Ap","Bm","Bp","Cm","Cp");

if ($network eq "u") {

@parameters=("run","slablength","minislablength","slabvol","dnaA","dnaB","dnaC","X0","Y0","KassX","KassY","ktransc","ktransl","kdegr","kdegp","kX","DX0","Dres","Dm","Dp","timestep","runtime","sampleperiod","graph");

@default=(0,2000,200,6e-6,1e-7,5e-9,3.5e-9,1e-6,1e-6,250,250,1e-2,1e-2,0,0,1e-3,25000,80,1000,1000,.1,1200,100);

} elsif ($network eq "r") {

@parameters=("run","slablength","minislablength","slabvol","dnaA","dnaB","dnaC","X0","Y0","KassX","KassY","KassAp","KassBp","ktransc","ktransl","kdegr","kdegp","kX","DX0","Dres","Dm","Dp","timestep","runtime","sampleperiod","graph");

@default=(0,2000,200,6e-6,1e-7,5e-9,3.5e-9,1e-6,1e-6,250,250,1e4,500,1e-2,1e-2,0,0,1e-3,25000,80,1000,1000,.1,1200,100);

} elsif ($network eq "m") {

@parameters=("run","slablength","minislablength","slabvol","dnaA","dnaB","dnaC","X0","Y0","KassX","KassY","KassAp","KassBpA","KassBpB","KassBpC","KassCp","ktransc","ktransl","kdegr","kdegp","kX","DX0","Dres","Dm","Dp","timestep","runtime","sampleperiod","graph");

@default=(0,2000,200,6e-6,1e-7,5e-9,3.5e-9,1e-6,1e-6,250,250,1e4,38.5,500,250,77,1e-2,1e-2,0,0,1e-3,25000,80,1000,1000,.1,1200,100);

} elsif ($network eq "c") {

@parameters=("run","slablength","minislablength","slabvol","dnaA","dnaB","dnaC","X0","Y0","KassX","KassY","ktransc","ktransl","kdegr","kdegp","kX","DX0","Dres","Dm","Dp","timestep","runtime","sampleperiod","graph");

@default=(0,2000,200,6e-6,1e-7,5e-9,3.5e-9,1e-6,1e-6,250,250,1e-2,1e-2,0,0,1e-3,25000,80,1000,1000,.1,1200,100);

}

\# useful accessory variable: number of parameters excluding output options (graph and sampleperiod are included(=2))

#$basicparam=$#parameters-$#species;

$basicparam=$#parameters;

foreach $name (@species) { $output="output".$name; push (@parameters,$output) };

print "\n\nparameter values are needed to start simulating........\n\nplease type:\n\n";

print "\t\tp or filename\t\tto read parameter values from a file\n\n";

print "\t\td\t\t\tto do a run with the default values\n\n";

print "\t\t[enter]\t\t\tto enter values manually\n\nchoice:\t";

chomp($runmode=<STDIN>);

\# test if something has been entered

\# if so, test whether it is 'd'(=default)

\# if not 'd', expect either p or a filename and open the files

\# if nothing, prompt user for values

if ($runmode)

{

if ($runmode eq "d")

{

print "\nloading default parameter values............\n\n";

if ($network eq "c") { print "\nusing connectivity matrix of repressed system\n" ; $network="r";	}

for $k (0..$#default) { $$values[$k][$sets]=${$parameters[$k]}=$value=$default[$k]; print "$parameters[$k]:$value\t" };

$sets=1;

}

else

{

if ($runmode eq "p") { $runmode="param.txt"; print "\nloading parameter values from param.txt...............\n\n"};

open (FILE,"<$runmode") or die "\n\nwarning!\ncould not open \"$runmode\" :\n$!";

$sets=0;

while (<FILE>)

{

chomp;	# remove trailing newline characters

s/#.*//;	# ignore comments by erasing them

next if /^(\s)*$/;	# skip blank lines

@list=split(/\t/,$_);	# read values and assign them to the proper variables

$size=$#list+1; $min=$basicparam-3;

if ($size<$min)

{

print "\t\twarning!\n\nthe file read contains $size values\n";

print "the minimal set needed to carry out a simulation is $min entries...\n\n";

print "\t\tprogram exiting.....please correct\n\n";

exit

}

elsif ($size>$min)

{

for $c (0..$#species) { if ($list[$basicparam+$c]==1) { push (@names,$species[$c]) } }

}

print "\n\nset:\t"; print $sets+1;

print "\tparameter values:\n\n";

for $k (0..$#list) { $$values[$k][$sets]=${$parameters[$k]}=$value=$list[$k]; print "$parameters[$k]:$value\t" };

$sets++;

}

close FILE;

print "\n\nfound $sets parameter set(s) in the file\n";

if ($network eq "c") {

print "\nplease specify location and name of connectivity data:\n\n\t(press enter if it is mat.txt):\t";

$mat=<STDIN>; if ($mat="\n") { $mat="mat.txt" };chomp($mat);

open (MATFILE,"<$mat") or die "\n\nwarning!\ncould not open \"$mat\" :\n$!";

$num=2;

while (<MATFILE>)

{

chomp;

@matlist=split(/\t/,$_);	# read values and assign them to the proper variables

for $i (0..$#matlist) { $$Kass[$num][$i]=$matlist[$i] }

if ($#matlist!=2) { die "\n\nplease check matrix file for incorrect size : \n\n\tmatrix should be 3 by 3, with columns corresponding to genes A,B and C and lines corresponding to transcription factors Ap,Bp, and Cp\n"; }

$num++;

}

if ($num!=5) { die "\n\nplease check matrix file for incorrect size : \n\n\tmatrix should be 3 by 3, with columns corresponding to genes A,B and C and lines corresponding to transcription factors Ap,Bp, and Cp\n"; }

}

}

}

else

{

print "\nmanual entry of parameter values....:\n\n";

\# ask user to type all parameter values (default values are assigned when none is specified expect for the output details)

for $k (0..$basicparam)

{

print "$parameters[$k]:\t";

chomp($value=<STDIN>);

if (!$value&&$value ne "0") {$value=$default[$k]; print "\t$value\n" };

$$values[$k][0]=${$parameters[$k]}=$value;

}

$sets=1;

if ($network eq "c") {

print "\nplease specify location and name of connectivity data:\n\n\t(press enter if it is mat.txt):\t";

$mat=<STDIN>; if ($mat="\n") { $mat="mat.txt" };chomp($mat);

open (MATFILE,"<$mat") or die "\n\nwarning!\ncould not open \"$mat\" :\n$!";

$num=2;

while (<MATFILE>)

{

chomp;

@matlist=split(/\t/,$_);	# read values and assign them to the proper variables

for $i (0..$#matlist) { $$Kass[$num][$i]=$matlist[$i] }

if ($#matlist!=2) { die "\n\nplease check matrix file for incorrect size : \n\n\tmatrix should be 3 by 3, with columns corresponding to genes A,B and C and lines corresponding to transcription factors Ap,Bp, and Cp\n"; }

$num++;

}

if ($num!=5) { die "\n\nplease check matrix file for incorrect size : \n\n\tmatrix should be 3 by 3, with columns corresponding to genes A,B and C and lines corresponding to transcription factors Ap,Bp, and Cp\n"; }

}

}

##########################################################

\# default connectivity matrixes, corresponding to different networks

##########################################################

\# values for a given network are stored in a 3 by 5 matrix called %Kass where columns are promoters (A,B,C)and lines are transcription regulators (X,Y,A,B,C).

$$Kass[0][0]=$KassX;$$Kass[0][1]=$KassX;$$Kass[0][2]=0;

$$Kass[1][0]=0;$$Kass[1][1]=0;$$Kass[1][2]=$KassY;

if ($network eq "u") {

print "unrepressed system";

$$Kass[2][0]=0;$$Kass[2][1]=0;$$Kass[2][2]=0;

$$Kass[3][0]=0;$$Kass[3][1]=0;$$Kass[3][2]=0;

$$Kass[4][0]=0;$$Kass[4][1]=0;$$Kass[4][2]=0;

}

elsif ($network eq "r") {

print "repressed system";

$$Kass[2][0]=0;$$Kass[2][1]=$KassAp;$$Kass[2][2]=$KassAp;

$$Kass[3][0]=0;$$Kass[3][1]=$KassBp;$$Kass[3][2]=$KassBp;

$$Kass[4][0]=0;$$Kass[4][1]=0;$$Kass[4][2]=0;

}

elsif ($network eq "m") {

print "mutually repressed system";

$$Kass[2][0]=0;$$Kass[2][1]=$KassAp;$$Kass[2][2]=$KassAp;

$$Kass[3][0]=$KassBpA;$$Kass[3][1]=$KassBpB;$$Kass[3][2]=$KassBpC;

$$Kass[4][0]=$KassCp;$$Kass[4][1]=$KassCp;$$Kass[4][2]=0;

}

elsif ($network eq "c") {

print "custom system\n"; }

print " \nconnectivity matrix :\n$$Kass[2][0]\t$$Kass[2][1]\t$$Kass[2][2]\n$$Kass[3][0]\t$$Kass[3][1]\t$$Kass[3][2]\n$$Kass[4][0]\t$$Kass[4][1]\t$$Kass[4][2]\n\n";

#####################################################

\# finished loading parameter values................

#####################################################

#####################################################

\# if necessary, prompt user for run options........

#####################################################

\# the species for which to output results are the same for all sets in a same run of simul.pl

$s=0;

\# when no sampletime is specified, default is to output concentrations once at the end of the simulation. ie sampleperiod=runtime

if (!$$values[$basicparam-2])

{

${$parameters[$basicparam-2]}=$runtime;

print "\nsampleperiod: $runtime\n";

}

\# check if any output has been specified

$flag1=0;

for $a ($basicparam..$#parameters)

{

if ($$values[$a][$s]==1) {$flag1=1};

}

if ($flag1!=1)

{

\# prompt user for output details

$test=0;

print "\n\nspecies to output (type their names, separated by commas) ?\n\n";

while ($test==0)

{

print "full set of species available for this system is X,Y,Am,Ap,Bm,Bp,Cm,Cp\n\n";

print "default output: A, B and C protein concentrations\n\n";

print "choice:\t";

chomp($names=<STDIN>);

if ($names)

{

@names=split(/,/,$names);

\# add to run specifications

$flag2=0;

for $k (0..$#names)

{

for $j (0..$#species)

{

if ($names[$k] eq $species[$j]) {$$values[$basicparam+$j][$s]=1;$flag2++};

}

}

if ($flag2==($#names+1)) {$test=1}

else

{

print "\n\nwarning! not all names are correct,please type them again.............\n\n";

for $i ($basicparam..$#parameters) {$$values[$i][$s]=0}; # clear correct entries

}

}

else

{

$test=1;

print "\tAp,Bp,Cp"; @names=("Ap","Bp","Cp");

for $b ($basicparam..$#parameters) { $$values[$b][$s]=$default[$b]};

}

}

\# outputs are specified

}

#########################################################################################################################################

##############

\# create output files and folders : one to store parameter values used to carry out the simulation

\# and if a timecourse is run : 2 files per desired species (one to store concentrations, one with concentrations normalized over all minislabs)

\# if a single time is output, only 2 files are created, one for concentrations of all species, one for normalized values of these concentrations..

#########################################################################################################################################

##############

$test=0;

while ($test==0)

{

print "\n\n\nplease provide a name for the folder to place simulation results :\n\n(<ENTER> for default) :\t";

chomp($results=<STDIN>);

if ($results)

{

$outputfolder=$results;

$address=$outputfolder;

}

else {

$outputfolder="X";

$outputfolder.=int(rand(10000));

$destination="simul-results";

$dest1=$destination."/single-runs";

$dest2=$destination."/multiple-runs";

$dest3=$dest1."/timecourse";

$dest4=$dest2."/timecourse";

$dest5=$dest1."/single-time";

$dest6=$dest2."/single-time";

if (!-e $destination) { mkdir ($destination,777) or print "\nwarning: could not create simul-results folder to save

results...\n"; }

if (!-e $dest1) { mkdir ($dest1,777) or print "\nwarning: could not create folder to save results...\n"; }

if (!-e $dest2) { mkdir ($dest2,777) or print "\nwarning: could not create folder to save results...\n"; }

if (!-e $dest3) { mkdir ($dest3,777) or print "\nwarning: could not create folder to save results...\n"; }

if (!-e $dest4) { mkdir ($dest4,777) or print "\nwarning: could not create folder to save results...\n"; }

if (!-e $dest5) { mkdir ($dest5,777) or print "\nwarning: could not create folder to save results...\n"; }

if (!-e $dest6) { mkdir ($dest6,777) or print "\nwarning: could not create folder to save results...\n"; }

if ($sets==1) {$folderA="/single-runs"} else {$folderA="/multiple-runs"}

if ($$values[24][$s]!=$$values[23][$s]) {$folderB="/timecourse/"} else {$folderB="/single-time/"}

$address=$destination.$folderA.$folderB.$outputfolder;

}

if (-e $address)

{

print "\nwarning!\n\nthis folder already exists. do you want to overwrite the files ?(y/n)\t";

chomp($answer=<STDIN>);

if ($answer eq "y") {$test=1}

elsif ($answer eq "n") {$test=0 }

}

else {	mkdir ($address,777) or die "\n\nwarning!\ncould not open \"$address\" :\n$!"; $test=1}

}

print "\n\noutputting files to '$address'\n";

open (param,">>$address/param.txt");

if ($$values[25][$s]) {open (graph,">>$address/graph.txt")}

\# where a single run with a single time is chosen for output, dump all the results in the same file....

if ($sets==1 && $sampleperiod==$runtime) { open DATA,">>$address/data.xls"; open NORM, ">>$address/normdata.xls" }

else

{

foreach $name (@names)

{

open ($name,">>$address/$name.xls");

$norm="norm".$name;

open ($norm,">>$address/norm$name.xls");

}

}

################################

\# loop through simulation sets

################################

\# for each run, the full set k of values needed to simulate the system are stored on row k of @@values (array of arrays)

for $s (0..$sets-1)

{

###########################################

\# write all values to the parameter file

###########################################

for $v (0..$#default)

{

print param "$$values[$v][$s]\t";

}

print param "\n";

###########################################

\# write run number in output files

###########################################

foreach $name (@names)

{

$rout=$$values[0][$s];

print $name " results for run $rout\n";

$norm="norm".$name;

print $norm " results for run $rout\n";

}

#####################################################

\# assign current set of values to parameter names:

#####################################################

for $param (0..$#parameters) {$look=${$parameters[$param]}=$$values[$param][$s];

#print "$look\n";

} ;

#######################

\# accessory variables

#######################

$f=$slablength/$minislablength;	# number of minislabs per slab9/23/2002

$stepnumber=int($runtime/$timestep)+1;	# number of simulation steps per run, rounded to cover whole simulation time

$simsample=int($sampleperiod/$timestep);	# number of simulation steps to perform before outputting next set of results

$diffM=$Dm*$timestep/$minislablength/$minislablength;	# general diffusion factor for mRNAs

$diffP=$Dp*$timestep/$minislablength/$minislablength;	# general diffusion factor for proteins

if ($kX==0)

{

$DX=$DX0;	# assigns fixed value to DX if decay rate is 0

$diffX=$DX*$timestep/$minislablength/$minislablength;	# diffusion factor for T7

}

#####################################

\# initial values of model variables

#####################################

\# each concentration is stored in 2 arrays : one for the present timestep and one for the previous (all others need not be stored)

\# this @ is initialized at zero

foreach $name (@species)

{

for $minislab (1..9*$f) {${$name}[$minislab]=0}

}

\# specification of all non-zero values at initial time

\# the initial distribution of X and Y is hard coded in the following lines...maybe later, we can make this more flexible...

\# X0 is the amount of X to be found in slab 1 as well as in slab 9, Y0 of Y is evenly distributed among all 9 slabs

\# initial concentrations of proteins X and Y in all slabs in umol : estimated from Bradford test : 1pmol/ul of stock

\# deposit is .5ul of T7 at each end slab, .5ul total of SP6 in whole chamber

for $minislab (1..$f)

{

$X[$minislab]=$X0/2/$slabvol;

}

for $minislab (8*$f+1..9*$f)

{

$X[$minislab]=$X0/2/$slabvol;

}

for $minislab (1..9*$f)

{

$Y[$minislab]=$Y0/9/$slabvol;

}

#####################

\# simulation steps

#####################

$runnumber++;

print "run: $runnumber\n";

for $simtime (0..$stepnumber-1)

{

\# in case of exponentially decaying function for T7, update value

if ($kX!=0)

{

$DX=($DX0-$Dres)*exp(-$kX*$simtime)+$Dres;

$diffX=$DX*$timestep/$minislablength/$minislablength;	# apparent diffusion factor for T7 modeled as exponentially decaying value

}

\# loop through all species

foreach $name (@species)

{

\# special cases : assign proper diffusion factor to proper species type, X and Y production terms are 0

if ($name =~ /p/) {$diff=$diffP} else {$diff=$diffM};

if ($name eq "X") { $diff=$diffX; $prod=0 };

if ($name eq "Y") { $prod=0	}

\# loop through all minislabs

for $minislab (1..9*$f)

{

\#	print "time: $simtime\tname: $name\tnetwork: $network\tprod: $prod\n";

$Aprot=$Ap[$minislab];

$Bprot=$Bp[$minislab];

$Cprot=$Cp[$minislab];

$slab=int(($minislab-1)/$f)+1; # keeps track of slab number

\# proteins

if ($name =~ /X|Y/) { $deg=-$timestep*$kdegp*${$name}[$minislab]	}

if ($name =~ /[ABC][p]/)

{

$Rname=$name;

$Rname =~ s/p/m/;

$prod=$ktransl*${$Rname}[$minislab] ; #print "mslab $minislab : $prod\t";

$deg=-$timestep*$kdegp*${$name}[$minislab];

}

\# rnas

if ($name eq "Am")

{

\# transcription of A only occurs in the slabs containing dna A, ie hard-wired as slabs 1 and 9

if ($slab =~ /1|9/ )

{

$pol=$X[$minislab];

\# $ktransc=$ktransc*($dnaA/$dnaA+$dnaB);

if ($network eq "u" or $network eq "r") {$prod=$timestep*$ktransc*$dnaA/$slabvol*$pol*$$Kass[0][0]/(1+$pol*$$Kass[0][0]);}

elsif ($network eq "m")

{$prod=$timestep*$ktransc*$dnaA/$slabvol*$pol*$$Kass[0][0]/(1+$pol*$$Kass[0][0]+$Bprot*$$Kass[3][0]+$Cprot*$$Kass[4][0])}

elsif ($network eq "c")

{$prod=$timestep*$ktransc*$dnaA/$slabvol*$pol*$$Kass[0][0]/(1+$pol*$$Kass[0][0]+$Aprot*$$Kass[2][0]+$Bprot*$$Kass[3][0]+$Cprot*$$Kass[4][0])}

}

else {$prod=0};

$deg=-$timestep*$kdegr*${$name}[$minislab];

}

if ($name eq "Bm")

{

\# if ($slab =~ /1|9/ )	{$ktransc=$ktransc*($dnaB/$dnaA+$dnaB)} ;

$pol=$X[$minislab];

if ($network eq "u") {$prod=$timestep*$ktransc*$dnaB/$slabvol*$pol*$$Kass[0][1]/(1+$pol*$$Kass[0][1]);}

elsif ($network eq "r") {$prod=$timestep*$ktransc*$dnaB/$slabvol*$pol*$$Kass[0][1]/(1+$pol*$$Kass[0][1]+$Aprot*$$Kass[2][1]+$Bprot*$$Kass[3][1]); } #print "\tmslab $minislab:$prod";}

elsif ($network eq "m" || $network eq "c") {$prod=$timestep*$ktransc*$dnaB/$slabvol*$pol*$$Kass[0][1]/(1+$pol*$$Kass[0][1]+$Aprot*$$Kass[2][1]+$Bprot*$$Kass[3][1]+$Cprot*$$Kass[4][1])};

$deg=-$timestep*$kdegr*${$name}[$minislab];

}

if ($name eq "Cm")

{

$pol=$Y[$minislab];

if ($network eq "u") {$prod=$timestep*$ktransc*$dnaC/$slabvol*$pol*$$Kass[1][2]/(1+$pol*$$Kass[1][2]);}

elsif ($network eq "r") {$prod=$timestep*$ktransc*$dnaC/$slabvol*$pol*$$Kass[1][2]/(1+$pol*$$Kass[1][2]+$Aprot*$$Kass[2][2]+$Bprot*$$Kass[2][2])}

elsif ($network eq "m") {$prod=$timestep*$ktransc*$dnaC/$slabvol*$pol*$$Kass[1][2]/(1+$pol*$$Kass[1][2]+$Aprot*$$Kass[2][2]+$Bprot*$$Kass[3][2])}

elsif ($network eq "c") {$prod=$timestep*$ktransc*$dnaC/$slabvol*$pol*$$Kass[1][2]/(1+$pol*$$Kass[1][2]+$Aprot*$$Kass[2][2]+$Bprot*$$Kass[3][2]+$Cprot*$$Kass[4][2])}

$deg=-$timestep*$kdegr*${$name}[$minislab];

}

\# local concentration gradient

\# minislabs at each end have a single neighbour with which to exchange molecules

if ($minislab==1) { $grad=${$name}[$minislab+1]-${$name}[$minislab] }

elsif ($minislab==9*$f) { $grad=${$name}[$minislab-1]-${$name}[$minislab] }

else { $grad=${$name}[$minislab-1]+${$name}[$minislab+1]-2*${$name}[$minislab] };

\# update formula

$next{$name}[$minislab]=${$name}[$minislab]+$diff*$grad+$deg+$prod;

if ($simtime%$simsample==0) {$conc{$name}=$conc{$name}+$next{$name}[$minislab]};

\# next minislab

}

\# next species

}

\# output the data if elapsed time is a non-zero multiple of sampleperiod or if this step completes the run

unless ($simtime==0)

{

if (($simtime%$simsample==0)|($simtime==$stepnumber-1))

{

$realtime=$simtime*$timestep;

print "time:$realtime\n";

\# write concentrations and normalized concentrations to the output files

foreach $name (@names)

{

for $mslab (1..9*$f)

{

if ($sets==1 && $sampleperiod==$runtime)

{

print DATA "${$name}[$mslab]\t";

$norm="norm".$name;

$normvalue=${$name}[$mslab]/$conc{$name};

print NORM "$normvalue\t";

}

else

{

print $name "${$name}[$mslab]\t";

$norm="norm".$name;

$normvalue=${$name}[$mslab]/$conc{$name};

print $norm "$normvalue\t";

}

}

if ($sets==1 && $sampleperiod==$runtime)

{

print DATA "\n";

print NORM "\n";

}

else

{

print $name "\n";

print $norm "\n";

}

}

\# flush %conc

map { $conc{$_} = 0 } keys %conc ;

}

}

\# values in present array are replaced by those in next....

foreach $name (@species) { for $mslab (1..9*$f) { ${$name}[$mslab]=$next{$name}[$mslab]; } }

\# proceed to next step in simulation

}

\# proceed to next set

}

\# close all output files

if ($sets==1 && $sampleperiod==$runtime) { close DATA; close NORM };

foreach $name (@names)

{

close $name;

$norm="norm".$name;

close $norm;

}

$totaltime=time()-$^T;

print "\nexecution time was: $totaltime seconds\n";

\# wait a second before displaying new prompt

\# sleep 1;

###########################

\# end of file

###########################
